# Tissue-wide genetic and cellular landscape shapes the execution of sequential PRC2 functions in neural stem cell lineage progression

**DOI:** 10.1126/sciadv.abq1263

**Published:** 2022-11-02

**Authors:** Nicole Amberg, Florian M. Pauler, Carmen Streicher, Simon Hippenmeyer

**Affiliations:** Institute of Science and Technology Austria (ISTA), Am Campus 1, 3400 Klosterneuburg, Austria.

## Abstract

The generation of a correctly sized cerebral cortex with all-embracing neuronal and glial cell–type diversity critically depends on faithful radial glial progenitor (RGP) cell proliferation/differentiation programs. Temporal RGP lineage progression is regulated by Polycomb repressive complex 2 (PRC2), and loss of PRC2 activity results in severe neurogenesis defects and microcephaly. How PRC2-dependent gene expression instructs RGP lineage progression is unknown. Here, we use mosaic analysis with double markers (MADM)–based single-cell technology and demonstrate that PRC2 is not cell-autonomously required in neurogenic RGPs but rather acts at the global tissue-wide level. Conversely, cortical astrocyte production and maturation is cell-autonomously controlled by PRC2-dependent transcriptional regulation. We thus reveal highly distinct and sequential PRC2 functions in RGP lineage progression that are dependent on complex interplays between intrinsic and tissue-wide properties. In a broader context, our results imply a critical role for the genetic and cellular niche environment in neural stem cell behavior.

## Teaser

The genetic state of cellular environment shapes PRC2-mediated neural stem cell behavior in the developing cerebral cortex.

## INTRODUCTION

The mammalian neocortex is composed of a rich variety of cell types and regulates critical cognitive and behavioral functions in the brain. Most neocortical cells include distinct excitatory and inhibitory neuronal classes and glial cell types (*1*). During cortical development, excitatory projection neurons and glial cells are generated from radial glial progenitors (RGPs) in a temporal sequential manner (*2*–*6*). RGP lineage progression in the developing embryonic cortex occurs in three major consecutive stages: (i) symmetric expansion of RGP pool, (ii) asymmetric neurogenic phase, and (iii) initiation of glial cell production (*6*, *7*). RGPs also generate a variety of distinct postnatal lineages (besides glial cells) including ependymal cells or postnatal neural stem cells (*8*). Recent quantitative single RGP cell lineage tracing by using mosaic analysis with double markers (MADM) revealed that overall RGP proliferation behavior is stereotyped and predictable to a large extent (*9*–*12*). In other words, RGPs undergo a defined series of symmetric proliferative divisions before switching to asymmetric neurogenic division. Once in asymmetric division mode, individual neurogenic RGPs generate a unitary output of about eight to nine neurons before transiting to glia production with about one in six RGPs proceeding to generate glial cells (*2*, *13*). Although the above inaugural quantitative framework has been established at the single-cell level, the precise cellular and molecular mechanisms regulating the orderly RGP lineage progression are not well understood.

At the individual cell level, RGPs appear to undergo a series of molecular identity and/or transcriptional state changes correlating with lineage progression and the generation of correctly specified progeny (*14*–*16*). In general, the transcriptional landscape in stem cell lineage progression is tightly regulated by epigenetic mechanisms (*17*) including Polycomb repressive complex 2 (PRC2)–mediated posttranslational chromatin modifications. PRC2 consists of three core subunits that are essential for proper catalytic activity and transcriptional repression in vivo: embryonic ectoderm development (EED), enhancer of zeste 2 (EZH2) or its homolog EZH1, and suppressor of zeste 12 (SUZ12) (*18*). Both EZH2 and EZH1 contain a conserved SET domain capable of catalyzing the mono-, di-, and trimethylation of lysine-27 of histone H3 (H3K27) (*18*). H3K27me3 is recognized by PRC1, which catalyzes the monoubiquitinylation of lysine-119 of histone H2A (H2AK119ub) and thereby ultimately induces transcriptional silencing (*19*, *20*). Ablation of any one of the PRC2 core components *Eed*, *Ezh2*, or *Suz12* results in complete disruption of the complex and subsequent loss of H3K27me3 (*21*, *22*), disrupting embryonic development (*23*). In effect, full gene knockout of either, *Eed*, *Ezh2*, or *Suz12* results in early embryonic lethality (*18*).

PRC2 activity is critical for the orchestration of cortical projection neuron and glial cell generation (*16*, *24*–*27*). Conditional loss [conditional knockout (cKO)] of PRC2 function by ablation of *Ezh2* (*26*) or *Eed* (*16*) in *Emx1^+^* RGPs results in marked thinning of the developing cortical wall with concomitant microcephaly. In correlation with the strong microcephaly observed in *Eed* cKO, RGPs seem to show accelerated temporal progression with shortened neurogenic period (*16*). At later developmental stages, the absence of PRC2 in RGPs was shown to affect the neurogenic-to-gliogenic switch (*25*, *26*). However, the exact function of PRC2 in astrocyte generation is still under debate because Hirabayashi *et al.* (*25*) observed delayed gliogenesis in *Ezh2* cKO, whereas Pereira *et al.* (*26*) reported precocious onset of gliogenesis. The discrepancy of the above findings may reflect a putative dual role for PRC2 in regulating major transitions in RGPs at both neurogenic and gliogenic competence levels, albeit concrete evidence for such mode of PRC2 action is currently lacking (*16*, *25*, *26*). In summary, on the basis of whole-tissue ablation, PRC2 has been proposed to exert several critical roles in cortical RGPs. How PRC2 regulates RGP proliferation behavior at the individual cell level in distinct stages along their lineage progression remains unknown.

Because of its cell-intrinsic mode of action, PRC2 activity is generally thought to be a cell-autonomous one. Yet, individual RGPs in vivo are embedded within the neocortical ventricular stem cell niche and thus operate in a complex cellular environment. Whether global tissue-wide properties shape the cell-autonomous PRC2 requirement or function in individual RGPs is currently unclear. Previous studies mainly used experimental paradigms with global and/or whole tissue-wide loss of PRC2 function, and analysis of RGP lineage progression lacked single-cell resolution. To overcome this limitation, here, we used MADM technology to analyze the functional requirement of PRC2 at the individual RGP cell level. MADM enables the generation of sparse mutant clones (*28*–*30*) and thus the study of true cell-autonomous PRC2 requirement. We contrasted the sparse PRC2 elimination with whole-tissue ablation paradigm and genetically dissected the interplay of PRC2 cell-autonomous and global tissue requirement. Contrary to its predicted essential function in embryonic neurogenic RGPs, we could show that PRC2 is not cell-autonomously required in the control of neurogenesis but rather plays a critical role at the global tissue-wide level. In contrast, at postnatal stages, PRC2 was cell-autonomously required for astrocyte production and maturation. Together, our data revealed distinct sequential, cell-autonomous, and global tissue-wide functional PRC2 requirements in RGP lineage progression during cortical development.

## RESULTS

### Global but not sparse KO of PRC2 results in diminished cortical projection neuron production and microcephaly

**Fig. 1.**
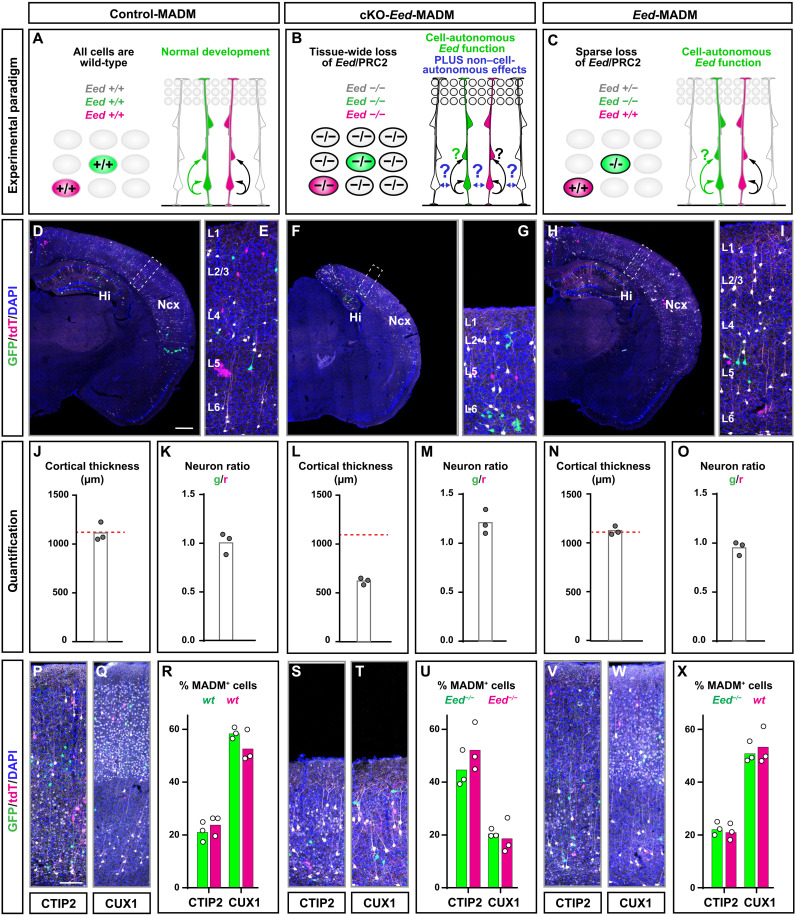
*Eed*/PRC2 is not cell-autonomously required in cortical neurogenesis. See also figs. S1 and S2. (**A** to **C**) Schematic overview of experimental paradigm and genotype of MADM-labeled cells in (A) wild-type MADM (control-MADM), (B) cKO of *Eed* in all cortical projection neurons (cKO-*Eed*-MADM), and (C) sparse genetic mosaic with *Eed* mutant cells labeled in green color (*Eed*-MADM). (**D** to **I**) Overview of MADM-labeling pattern in somatosensory cortex in (D) control-MADM, (F) cKO-*Eed*-MADM, and (H) *Eed*-MADM mice at P21. (E), (G), and (I) depict higher-resolution images of boxed areas in (D), (F), and (H) with indication of cortical layers. (**J** to **O**) Quantification of cortical thickness and g/r neuron ratio, respectively, in (J to K) control-MADM, (L and M) cKO-*Eed*-MADM, and (N and O) *Eed*-MADM. (**P** to **X**) Histological stainings of P21 brains for lower-layer marker CTIP2 and for upper-layer marker CUX1 and quantification of the percentage (%) of green and red MADM-labeled CTIP2^+^ and CUX1^+^ cells in (P to R) control-MADM, (S to U) cKO-*Eed*-MADM, and (V to X) *Eed*-MADM mice. Each individual data point represents one experimental animal. Data indicate mean ± SEM. Scale bars, 500 μm (D, F, and H) and 60 μm (E, G, I, P, Q, S, T, V, and W).

To obtain insights of PRC2 function in RGPs with single-cell resolution, we combined a floxed allele of the core subunit *Eed* [*Eed*-flox; (*31*)] with MADM cassettes located on chr.7 (MADM-7) (fig. S1) (*32*). In a first experiment, we conditionally removed *Eed* in a global tissue-wide manner and generated *Eed* cKO mice, with concomitant sparse MADM labeling, in combination with *Emx1*-Cre (*33*) (*MADM-7^GT,Eed/TG,Eed^;Emx1^Cre/+^*, abbreviated cKO-*Eed*-MADM). As expected and previously reported (*16*, *26*), cKO-*Eed*-MADM mice at postnatal day (P) 21 showed a strong decrease of neocortical thickness when compared to control-MADM (*MADM-7^GT/TG^;Emx1^Cre/+^*) animals (Fig. 1, A and B, D to G, J, and L). In both control-MADM (all cells *Eed*^+/+^) and cKO-*Eed*-MADM (all cells *Eed*^−/−^), the green [green fluorescent protein–positive (GFP^+^)] to red [tandem dimer tomato-positive (tdT^+^)] (g/r) ratio was ~1 reflective of the identical genotypes in GFP^+^ and tdT^+^ MADM-labeled cells (Fig. 1, K and M). The marked thinning of the neocortex in cKO-*Eed*-MADM reflects a decrease of neuron production, presumably because of accelerated lineage progression of neurogenic RGPs (*16*). Thus, assuming a purely cell-autonomous function of PRC2 in proliferating RGPs, we predicted a marked loss of *Eed* mutant cells in comparison to wild-type cells when analyzed in a sparse genetic mosaic as provided by the MADM system (Fig. 1C). To test the above, we generated mosaic *Eed*-MADM (*MADM-7^GT/TG,Eed^;Emx1^Cre/+^*) mice with sparse green GFP^+^ homozygous *Eed*^−/−^ mutant cells, yellow (GFP^+^/tdT^+^) heterozygous *Eed*^+/−^, and red (*tdT*^+^) *Eed*^+/+^ wild-type cells in an otherwise unlabeled heterozygous background (Fig. 1, C, H, and I, and fig. S1, D and E) (*34*). Contrary to our expectation, we found no reduction of green *Eed*^−/−^ mutant when compared to red *Eed*^+/+^ control neurons and thus a g/r ratio of ~1 at P21 (Fig. 1O). To validate the specificity of PRC2 activity elimination in green *Eed*^−/−^ mutant but not red *Eed*^+/+^ control cells in mosaic *Eed*-MADM mice, we stained for the PRC2-catalyzed histone mark H3K27me3 in E12.5 cortex (fig. S2). H3K27me3 signal, and thus PRC2 activity, was abolished in individual green *Eed*^−/−^ but not red *Eed*^+/+^ cells in mosaic *Eed*-MADM brains (fig. S2, C to F). In contrast and as expected, H3K27me3 was completely eliminated in all cells in cKO-*Eed*-MADM (fig. S2, G to J).

Next, we analyzed the generation of CTIP2^+^ (primarily lower layer V) relative to CUX1^+^ (upper layer IV-II) neurons. While we found very similar relative fractions of CTIP2^+^ and CUX1^+^ MADM-labeled cells in control-MADM and mosaic *Eed*-MADM (Fig. 1, P to R and V to X), the relative abundance of CTIP2^+^ MADM-labeled cells was significantly increased when compared to CUX1^+^ cells in cKO-*Eed*-MADM, in agreement with previous studies (Fig. 1, S to U) (*16*). We conclude that global KO of *Eed* in cKO-*Eed*-MADM leads to marked microcephaly because of significant reduction of upper layer IV-II relative to lower layer V projection neurons. In contrast, sparse deletion of *Eed* in genetic mosaic *Eed*-MADM paradigm did not affect projection neuron production and thus cortical size. Together, the above data suggested that *Eed* is not cell-autonomously required in cortical RGPs during neurogenic phase but rather at the global tissue-wide level.

### PRC2 function is not cell-autonomously required in cortical RGP-mediated projection neuron production

While the above analysis at the population level already provided an indication about cell-autonomy of *Eed* gene function based on sparseness of the genetic mosaic, conclusive assessment of PRC2 function at the individual RGP level is only possible using clonal analysis. Thus, we carried out MADM-based clonal analysis (*28*) to probe cell-autonomous PRC2 function at true single RGP cell level. To determine the neurogenic potential of individual mutant *Eed*^−/−^ RGPs, we pursued two MADM assays in combination with tamoxifen (TM)–inducible *Emx1^CreER^* driver (*35*).

**Fig. 2.**
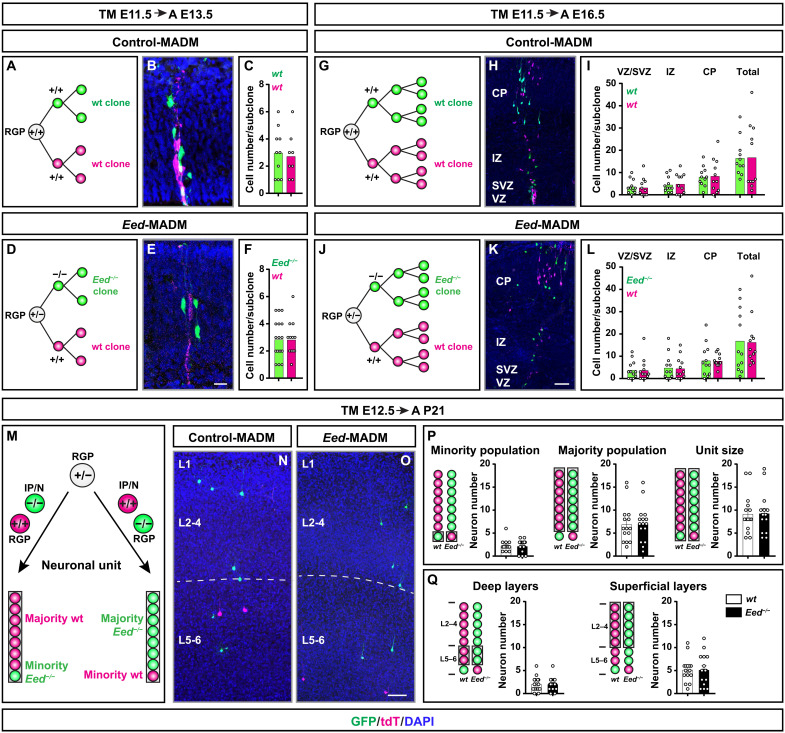
Functional analysis of *Eed*/PRC2 function during neurogenesis at single RGP clone level. (**A** to **F**) Experimental setup for embryonic MADM clones induced at E11.5 and analyzed at E13.5 with representative images and corresponding quantifications in (A to C) control-MADM and (D to F) *Eed*-MADM mice. (**G** to **L**) Experimental setup for embryonic MADM clones induced at E11.5 and analyzed at E16.5 with representative images and corresponding quantifications in (G to I) control-MADM and (J to L) *Eed*-MADM mice. (**M**) Experimental setup for MADM clones induced at E12.5 and analyzed at P21 to determine the neuronal unit size. Upon a MADM event in asymmetrically dividing RGPs, one daughter cell will become an intermediate progenitor or neuron (IP/N) and thus constitute the minority population of the clone, while the other daughter cell will remain RGP identity and produce the majority population of the clone. (**N** and **O**) Representative images of clones in (N) control-MADM and (O) *Eed*-MADM mice. (**P**) Quantification of the average neuron number within the minority population (left), the majority population (middle), and the total unit size (right) emerging from wild-type and *Eed*^−/−^ RGPs. (**Q**) Quantification of the average number of deep layer neurons (left) and superficial layer neurons (right) in wild-type and *Eed*^−/−^ MADM clones. Each individual data point represents one MADM clone. Data indicate mean ± SEM. Significance was determined using unpaired *t* test (C, F, P, and Q) or one-way ANOVA with Turkey’s multiple comparisons (I and L) using *P* < 0.05 as cut-off. Scale bars, 20 μm (B and E) and 50 μm (H, K, N, and O).

First, we analyzed RGPs in their symmetric proliferative mode (*9*). In the MADM context, the two daughter cells (and resulting subclones) from such symmetric proliferative RGP divisions will be labeled in different colors (red and green) and may inherit distinct genotypes (*28*). We first validated the approach because MADM clones using MADM-7 in combination with *Emx1^CreER^* driver had not been much analyzed previously. We thus first generated control *MADM-7^GT/TG^;Emx1^CreER^* and injected TM at embryonic day 11.5 (E11.5) when most RGPs still divide symmetrically (*9*). We quantified the cell numbers in the two red and green (both wild-type) subclones at E13.5 across the developing cortical wall (Fig. 2, A to C) and at E16.5, when most neurons have been generated (Fig. 2, G to I). As expected, the numbers of green and red wild-type cells at both E13.5 and E16.5 and in all analyzed zones was not significantly different (Fig. 2, C and I). Next, we generated *Eed*-MADM clones (*MADM-7^GT/TG,Eed^;Emx1^CreER^*) with similar TM injection/analysis regime as above but with the red subclone containing wild-type and the green subclone *Eed*^−/−^ mutant cells, respectively. At both E13.5 and E16.5 analysis time points, we counted similar cell numbers like in the above control-MADM clones. We could not detect significantly different numbers of red wild-type compared to green *Eed*^−/−^ mutant cells (Fig. 2, D to L). Thus, the proliferation potential of mutant *Eed*^−/−^ and wild-type subclones emerging from a single symmetrically dividing RGP appeared identical.

In the second assay, we quantified absolute neuron output of individual RGPs once they switched to asymmetric neurogenic proliferation mode (*9*). Previous MADM analysis, using MADM-11, has demonstrated unitary output of about eight to nine neurons from a single RGP (*9*, *10*, *36*). We injected TM at E12.5 and analyzed MADM clone composition at P21 (Fig. 2, M to O). We analyzed total unit size, majority population (larger subclone) and minority population (smaller subclone) (Fig. 2P), and distribution of MADM-labeled neurons in deep (VI-V) and superficial (IV-II) cortical layers (Fig. 2Q). In all quantifications, we could not detect a significant difference in the numbers of *Eed*^−/−^ mutant when compared to *Eed*^+/+^ control neurons, and the total unit size was about eight to nine neurons for each genotype. On the basis of the above results from single MADM clone analysis, we conclude that PRC2 function is not cell-autonomously required for cortical RGP-mediated neuron production.

### Distinct tissue-wide genetic environment in mosaic and global KO triggers differential ectopic gene expression in *Eed*^−/−^ mutant cells

**Fig. 3.**
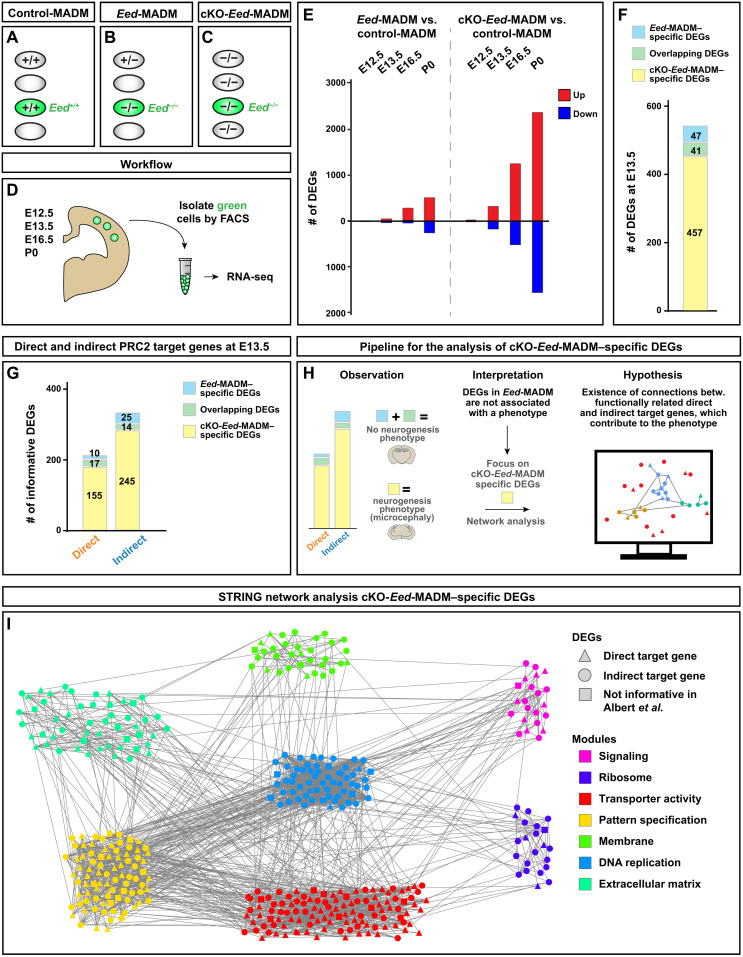
Deregulated genes specific to cKO-*Eed*-MADM are functionally connected and converge on cell cycle regulatory modules. See also figs. S3 and S4. (**A** to **C**) Schematic overview of experimental MADM paradigms and genotype of GFP^+^ cells in (A) control-MADM, (B) *Eed*-MADM, and (C) cKO-*Eed*-MADM. (**D**) Schematic overview of workflow for RNA-seq experiments at E12.5, E13.5, E16.5, and P0. (**E**) Number of DEGs (*P*_adj_ < 0.1, DESeq2) upon comparison of *Eed*^−/−^ mutant (*Eed*-MADM and cKO-*Eed*-MADM) cells to *Eed*^+/+^ cells in control-MADM at different developmental time points (E12.5, E13.5, E16.5, and P0). “Up” refers to genes up-regulated in *Eed*^−/−^ mutant cells, while “Down” refers to genes down-regulated in *Eed*^−/−^ mutant cells when compared to *Eed*^+/+^ cells. (**F**) Detailed analysis of E13.5 DEGs. Number of DEGs specific to *Eed*^−/−^ cells from *Eed*-MADM (light blue), specific to *Eed*^−/−^ cells from cKO-*Eed*-MADM (light yellow), and genes shared between both paradigms (light green). (**G**) Overlap analysis of E13.5 DEGs [same as in (F)] with H3K27me3 data (*39*). Number of direct and indirect PRC2 target genes specific to *Eed*^−/−^ cells from *Eed*-MADM (light blue), specific to *Eed*^−/−^ cells from cKO-*Eed*-MADM (light yellow), and genes shared between both paradigms (light green). Note that only genes informative in both datasets have been included in the analysis. For details, see text and Materials and Methods. (**H**) Schematic illustration of hypothesis-based STRING network analysis of cKO-*Eed*-MADM–specific DEGs. (**I**) STRING network showing protein-protein interactions of cKO-*Eed*-MADM–specific DEGs at E13.5. Triangles: direct PRC2 target genes, circles: indirect PRC2 target genes [same as in (G)], squares: genes not informative in (*39*). Note that DEGs group into functionally distinct modules as indicated by color code. For details, see text and Materials and Methods.

Although *Eed*^−/−^ cells in *Eed*-MADM and cKO-*Eed*-MADM paradigms had identical genotypes, they existed in distinct genetic and cellular environments. While in *Eed*-MADM the genetic background predominantly was *Eed*^+/−^ heterozygous with phenotypically regular sized cortex (Fig. 1N), the vast genetic landscape in cKO-*Eed*-MADM is *Eed*^−/−^ with massively diminished neuron numbers and much smaller overall cortex size (Fig. 1L). Given the marked phenotypic difference in individual MADM-labeled *Eed*^−/−^ mutant cells depending on their genetic and cellular environment, we measured global tissue-wide deregulation of gene expression to identify molecular correlates hinting at deregulated biological processes because of PRC2 loss of function. We used an established fluorescence-activated cell sorting (FACS)–based approach (*37*, *38*) to isolate GFP-labeled *Eed*^+/+^, *Eed*^+/−^, and *Eed*^−/−^ mutant cells in a time course at E12.5 (onset of neurogenesis), E13.5 (early neurogenesis), E16.5 (end of neurogenesis), and P0 (start of gliogenesis) (Fig. 3, A to D). Next, we performed small sample Smart-Seq2–based bulk RNA sequencing (RNA-seq) followed by transcriptome analysis. We confirmed that *Eed* expression was efficiently depleted in *Eed*^−/−^ mutant cells in *Eed*-MADM and cKO-*Eed*-MADM at all time points (fig. S3, A and B). We then aimed to directly test whether variable global gene expression in mutant *Eed*^−/−^ cells correlated with the genetic/cellular environment and determined the quantitative difference in sparse mutant *Eed*-MADM when compared to the global tissue cKO-*Eed*-MADM. We identified differentially expressed genes (DEGs) of *Eed*^−/−^ cells between *Eed*-MADM and cKO-*Eed*-MADM. A relatively low number of DEGs was found at E12.5 (8) and E13.5 (95), but increasing numbers of DEGs were present at E16.5 (1564) and P0 (3324). DEGs showed temporal developmental specificity with limited overlap between the different time points (fig. S3C).

To gain more detailed insights into gene expression changes in mutant cells relative to control, we compared *Eed*^−/−^ cells from *Eed*-MADM and cKO-*Eed*-MADM with *Eed*^+/+^ cells in control-MADM. Similar to the initial analysis, we found increasing numbers of DEGs from E12.5 to P0 (Fig. 3E). Consistent with the well-described function of PRC2 in transcriptional repression, most DEGs were composed of up-regulated genes (Fig. 3E and fig. S3C) (*19*). The number of DEGs was significantly higher in *Eed*^−/−^ cells from cKO-*Eed*-MADM than from *Eed*-MADM at any given developmental time point (10-fold higher at E12.5 and ~5-fold higher at E13.5, E16.5, and P0) (Fig. 3E).

To test whether the progressive and strong increase of DEGs in cKO-*Eed*-MADM correlated with the phenotype in vivo, we performed a time-course analysis of the embryonic progenitor pool and neuronal output in control-MADM, *Eed*-MADM, and cKO-*Eed*-MADM by staining for progenitor markers SOX2 and TBR2 and immature neuron marker NEUROD2, respectively (fig. S4, A to O). Assessment of complete cortical thickness and ventricular zone thickness (fig. S4P) did not reveal differences between the three genetic paradigms at E12.5 and E13.5 (fig. S4, A to F, Q, and R). In accordance with a previous study reporting altered RGP behavior in *Eed* cKO mice (*16*), we found a significantly reduced ventricular zone thickness in cKO-*Eed*-MADM cortices from E14.5 onward (fig. S4, G to O and Q), followed by a significantly diminished overall cortical thickness from E15.5 (fig. S4, J to O and R).

On the basis of the above findings, we focused further gene expression analysis on E13.5, which is before the onset of a histological phenotype, while transcriptomic differences between *Eed*-MADM and cKO-*Eed*-MADM are already strongly evident. Overlap analysis of E13.5 DEGs revealed that 457 DEGs were specifically deregulated in cKO-*Eed*-MADM, while only 47 DEGs were specific to *Eed*-MADM and 41 DEGs were shared between both paradigms (Fig. 3F).

PRC2 is a direct transcriptional repressor via deposition of the H3K27me3 marks. We therefore sought to identify deregulation of genes directly caused by the lack of PRC2 (direct target genes) and gene expression changes caused downstream of direct targets (indirect targets). To this end, we used a published H3K27me3 chromatin immunoprecipitation–sequencing (ChIP-seq) dataset from purified cortical progenitor cells from E14.5 cortex (*39*). Genes reported to contain H3K27me3 marks and showing significant up-regulation were classified as direct target genes. All other significantly deregulated genes were classified as indirect targets (fig. S5A). Such analysis identified four times more direct target genes in *Eed*^−/−^ cells from cKO-*Eed*-MADM (172) than from *Eed*-MADM (27) (Fig. 3G). In line with the function of PRC2 as a transcriptional repressor, we found a larger number of up-regulated genes when direct target genes were compared to indirect targets (fig. S5B). Up-regulated DEGs were much more abundant in direct than in indirect targets (fig. S5B, 27 versus 11 in *Eed*-MADM and 172 versus 104 in cKO-*Eed*-MADM). Yet, we found a greater overlap of DEGs classified as direct PRC2 target genes (17 of 27 or 63% in *Eed*-MADM versus 17 of 155 or 11% in cKO-*Eed*-MADM) compared to indirect target genes (14 of 39 or 36% in *Eed*-MADM and 14 of 259 or 6% in cKO-*Eed*-MADM) (Fig. 3G). Together, our results demonstrate that the genetic constitution of the cellular environment strongly influences the amount of transcriptionally silenced direct PRC2 target genes.

### Whole-tissue KO of *Eed* leads to deregulation of strongly connected but functionally diverse gene modules

The above data suggested that cKO-*Eed*-MADM–specific DEGs alone could cause cKO-*Eed*-MADM–specific phenotypes for two reasons: (i) The number of specific direct target genes is more than six times higher in a mutant environment (172 in cKO-*Eed*-MADM versus 27 in *Eed*-MADM) (Fig. 3G) and (ii) *Eed*^−/−^ cells in cKO-*Eed*-MADM but not in *Eed*-MADM showed neurogenesis deficits. Signaling complexes perform their biological activity in the context of larger modules consisting of functionally related and interacting proteins (*40*). We thus tested whether cKO-*Eed*-MADM–specific DEGs contained modules that were sufficient to explain the altered RGP behavior and microcephaly phenotype. To do so, we made use of the rich information of protein-protein interactions (PPI) in the STRING databases (*40*). We used the 88 *Eed*-MADM–specific plus overlapping DEGs and the 457 cKO-*Eed*-MADM–specific DEGs as two separate inputs to the STRING analysis (Fig. 3H and fig. S5C). Notably, only proteins associated with our list of cKO-*Eed*-MADM–specific DEGs, but not sparse *Eed*-MADM–specific DEGs, formed a highly significant and densely connected network (PPI enrichment *P* value < 1 × 10^−16^) (fig. S5C). For further analysis, we focused on the largest cKO-*Eed*-MADM–specific connected subnetwork, consisting of 400 genes (see Materials and Methods). Clustering analysis of this network identified seven main modules of strongly connected genes (Fig. 3I). Gene Ontology (GO) analysis of genes in each of these seven modules revealed significant enrichment of diverse terms (data table S1). The top three enriched GO terms were used to label each module (Fig. 3I and fig. S5, D to F). One module held the potential to directly explain the microcephaly phenotype: “DNA replication.” We therefore considered this as a core module of the network. Note that the remaining six modules covered a broad range of biological processes and showed a varying degree of connection to the core module, indicating functional relationship (Fig. 3I and fig. S5D). The proportion of direct PRC2 target genes in each module was also diverse (fig. S5E). The core module contained the least proportion of direct PRC2 targets, whereas transporter activity, extracellular matrix, and pattern specification contained a high number of direct PRC2 target genes (Fig. 3I and fig. S5E). In summary, we concluded that a subset of highly connected cKO-*Eed*-MADM–specific DEGs were sufficient to explain the microcephaly phenotype.

### Whole-tissue but not sparse KO of *Eed* leads to progenitor proliferation defects through deregulation of multiple redundant gene modules

**Fig. 4.**
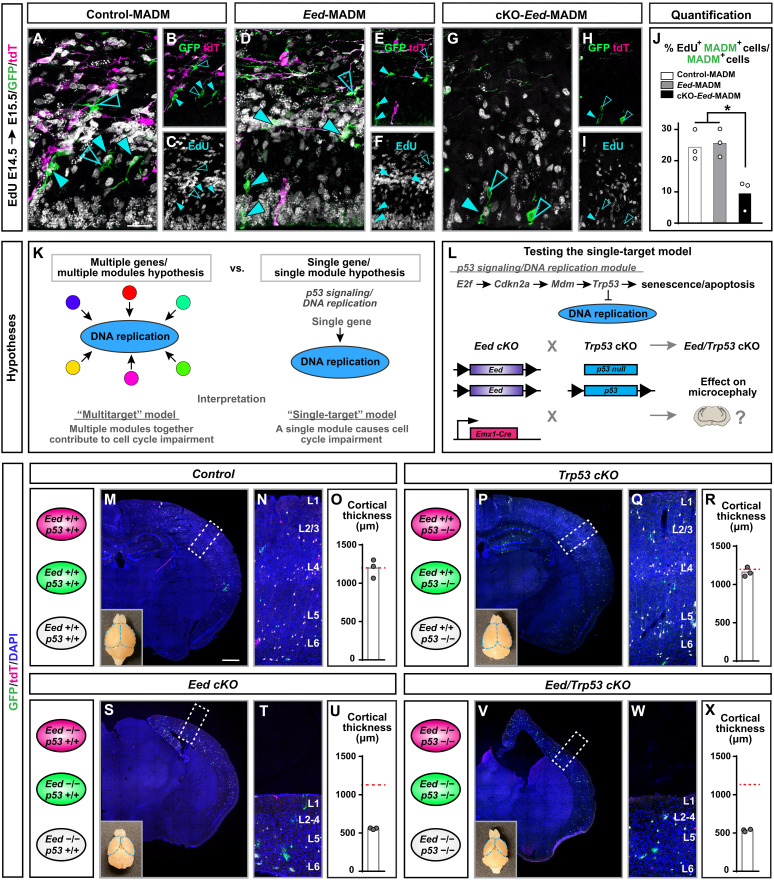
Proliferation deficits in *Eed*^−/−^ RGPs upon global tissue-wide loss of PRC2 activity occur independently of *Trp53* expression. See also fig. S5. (**A** to **I**) Immunofluorescence images of EdU staining after a 24-hour EdU pulse administered at E14.5 to (A to C) control-MADM, (D to F) *Eed*-MADM, and (G to I) cKO-*Eed*-MADM mice. (**J**) Percentage of EdU^+^ green MADM-labeled cells in (white) control-MADM, (gray) *Eed*-MADM, and (black) cKO-*Eed*-MADM. (**K**) Schematic summary of two hypotheses arising from STRING network analysis. (**L**) Schematic illustration of experimental approach to address the single module hypothesis by creating *Eed/Trp53* double cKO to genetically inactivate the p53 signaling/cell cycle module. (**M** to **X**) Schematics of cellular genotypes in respective experimental MADM paradigms and analysis of cortical thickness in (M to O) control, (P to R) *Trp53* cKO, (S to U) *Eed* cKO, and (V to X) *Eed/Trp53* double cKO mice at P21. (M, P, S, and V) Overview of MADM-labeling pattern in somatosensory cortex. (N), (Q), (T), and (W) depict higher-magnification images of boxed areas in (M), (P), (S), and (V) with indications of cortical layers. Lower left insets in (M), (P), (S), and (V) show macrographs of whole brains with respective genotypes at P21. (O, R, U, and X) Quantification of cortical thickness in (O) control, (R) *Trp53* cKO, (U) *Eed* cKO, and (X) *Eed/Trp53* double cKO mice. Each individual data point in (J), (O), (R), (U), and (X) represents one experimental animal. Statistics: (J) one-way ANOVA with Turkey’s multiple comparisons; *P < 0.05; **P < 0.01; ***P < 0.001. Data indicate mean ± SEM. Scale bars, 25 μm (A, D, and G), 50 μm (B, C, E, F, H, and I), 500 μm (M, P, S, and V), and 60 μm (N, Q, T, and W).

The STRING network suggested DNA replication as core module that was deregulated because of tissue-wide PRC2 deficiency. To directly test this possibility, we investigated progenitor proliferation potential in control-MADM, *Eed*-MADM, and cKO-*Eed*-MADM. We performed 24-hour Ethynyl deoxyuridine (EdU) pulse-chase experiments by injecting EdU into pregnant dams at E14.5 and analyzed the developing cortex at E15.5. Quantification of EdU^+^/GFP^+^ MADM-labeled cells showed that green RGPs in *Eed*-MADM (*Eed*^−/−^) displayed similar proliferation rates like green RGPs in control-MADM (*Eed*^+/+^) (Fig. 4, A to F and J). In contrast, green RGPs from cKO-*Eed*-MADM (*Eed*^−/−^) cortices showed 2.5-fold reduction in EdU incorporation (Fig. 4, G to J). Thus, our results confirm the findings from the STRING network analysis and identify progenitor proliferation as a core module in the microcephaly phenotype.

The topology of STRING network suggested a complex relationship of multiple deregulated gene modules culminating in proliferation defects. Following from this topology, it was possible that multiple modules operated redundantly (multiple module hypothesis; Fig. 4K, left). Such model implied that removal of a single module would not alter the overall phenotype. Alternatively, it was possible that a single module had a dominant effect on the phenotype (single module hypothesis; Fig. 4K, right). Removal of such predominant module was thus expected to rescue the microcephaly phenotype. Multiple lines of evidence supported the single-module hypothesis. For example, a number of genetic mutations in human and mouse lead to microcephaly because of cell cycle dysregulation and apoptosis. Defects in cell cycle can originate from the impairment of a plethora of different biological processes, including DNA replication that we also identified as a core module in the STRING network. Despite the diverse genetic causes, microcephaly phenotypes often converge to trigger p53 activation and apoptosis in dividing RGPs and/or nascent neurons. In such cases, genetic deletion of *Trp53* rescues the microcephaly phenotype (*41*–*44*). We noted the presence of p53-related GO terms in several modules of the STRING network (data table S1). We thus considered p53 signaling as a strong candidate for the single-module hypothesis (Fig. 4L).

To directly assay for apoptosis, we performed active caspase-3 stainings at E14.5 to determine the level of cell death. We detected a significant increase in the number of apoptotic cells (independent of MADM labeling) in cKO-*Eed*-MADM but not in *Eed*-MADM when compared to control-MADM cortices (fig. S6, A to G). We concluded that global tissue-wide KO of *Eed*, and thus loss of PRC2 activity, not only translated to reduced proliferative potential in RGPs but also increased apoptosis. In contrast, sparse or clonal ablation of *Eed* did not lead to RGP proliferation and/or survival deficits.

We proceeded with testing the “single-target hypothesis” by pursuing genetic epistasis experiments to inactivate the p53 signaling pathway in cKO-*Eed*-MADM cortices (Fig. 4L). We used cKO-Eed-MADM paradigm and *Trp53* deficiency models—*Trp53*-flox (*45*) and *Trp53* null allele (*46*)—to generate conditional whole-tissue (using *Emx1*-Cre) *Eed/Trp53* double mutants (cKO-*Eed*-MADM;*Trp53*-cKO, abbreviated *Eed*/*Trp53* cKO) (Fig. 4L). We first confirmed that *Trp53* deletion abolished apoptosis (i.e., presence of caspase-3^+^ cells) in developing cortex in *Eed*/*Trp53* cKO mice (Fig. S6, I to K). If microcephaly emerged in a predominantly p53-dependent manner in whole-tissue *Eed* cKO, we expected a partial or full rescue of microcephaly in *Eed*/*Trp53* cKO. Cortical thickness and severity of microcephaly in both *Eed* cKO and *Eed/Trp53* cKO mice, however, showed similar reduction (Fig. 4, M to X) when compared to control and *Trp53* cKO, respectively (Fig. 4, M to R). We stained for CUX1 and noticed that, in both *Eed* cKO and *Eed/p53* double cKO mice, CUX1^+^ upper layer neurons were similarly reduced (fig. S6, L and P). Together, our results indicated that elimination of the single *Trp53* gene module did not rescue the proliferation defect observed in *Eed* cKO mice. The data therefore support the multiple module hypothesis, where microcephaly originates from multiple, functionally redundant, deregulated gene modules.

### PRC2 cell-autonomously regulates astrocyte production and maturation in developing neocortex

Upon neurogenesis, RGPs obtain gliogenic potential and PRC2-mediated transcriptional repression has been implicated in cortical astrocyte generation, albeit controversial findings have been reported (*25*, *26*). We first determined whether PRC2 is active in developing astrocytes by performing H3K27me3 staining in immature astrocytes in control-MADM at P4, using *Eed*-MADM as negative control. We found that developing astrocytes in control-MADM mice showed pronounced H3K27me3 staining, while green *Eed*^−/−^ astrocytes in *Eed*-MADM cortices showed no detectable H3K27me3 signal (fig. S7, A to D).

**Fig. 5.**
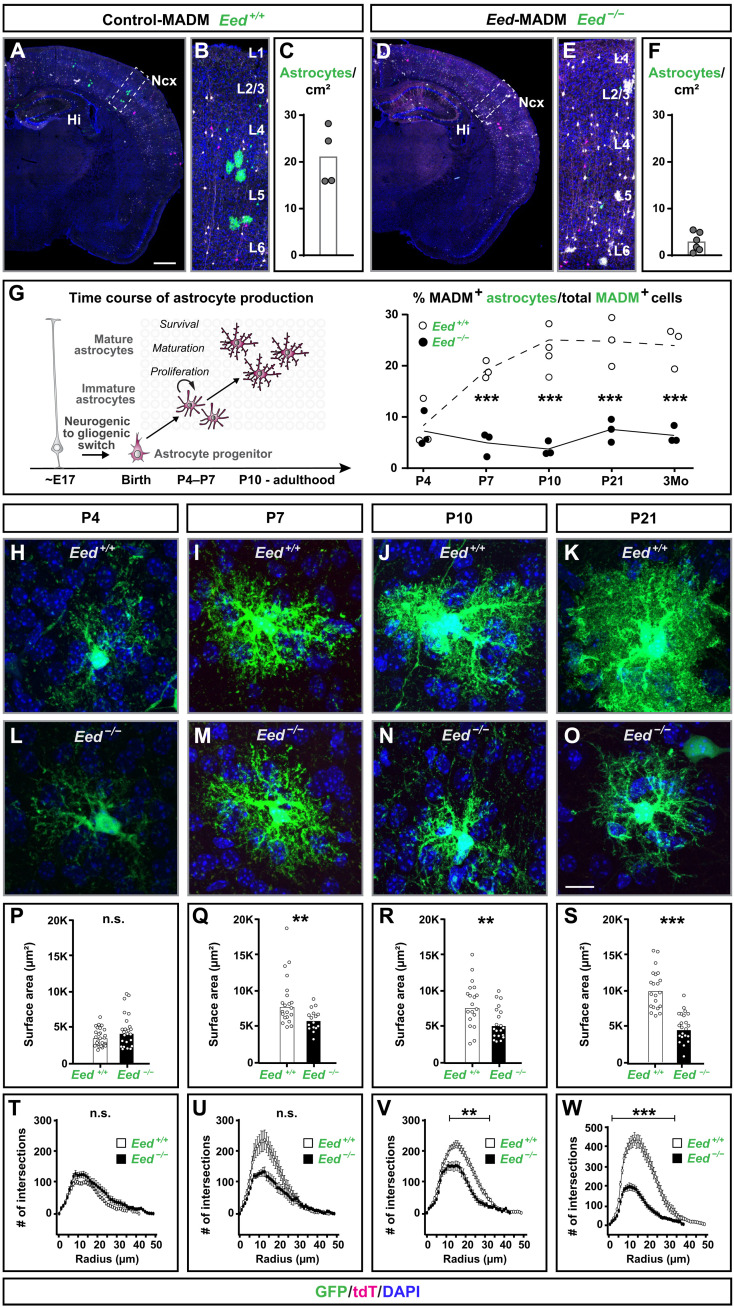
*Eed* is cell-autonomously required for cortical astrocyte production and maturation. See also fig. S6. (**A** to **F**) Overview of MADM-labeling pattern in the cortex in (A) control-MADM and (D) *Eed*-MADM mice at P21. (B and E) Boxed areas in (A) and (D) illustrating higher-resolution insets of the cortex with layer indications. (C and F) Quantification of astrocyte numbers with green MADM-labeled astrocytes/cm^2^ in (C) control-MADM and (F) *Eed*-MADM mice. (**G**) Percentage of green astrocytes in control-MADM (white) and *Eed*-MADM (black) at P4, P7, P10, P21, and 3 months. (**H** to **O**) High-resolution images of individual astrocytes from (H to K) control-MADM and (L to O) *Eed*-MADM. (**P** to **W**) Quantification of (P to S) surface area and (T to W) Sholl analysis of *Eed*^+/+^ (white) and *Eed*^−/−^ (black) astrocytes at (H, L, P, and T) P4, (I, M, Q, and U) P7, (J, N, R, and V) P10, and (K, O, S, and W) P21. (C, F, and G) Each individual data point represents one experimental animal. Data show mean ± SEM. (P to S) Each data point represents one individual astrocyte. Data show mean ± SEM. (T to W) Graphs show mean ± SEM. The number of astrocytes used for this analysis is equivalent to the number of astrocytes used in the respective surface area analysis. Statistics for (G) percentage of green astrocytes: two-way ANOVA with Sidak’s multiple comparisons test; *P < 0.05; **P < 0.01; ***P < 0.001. Statistics for (P to S) surface area: unpaired *t* test with *P < 0.05; **P < 0.01; ***P < 0.001. Statistics for (T to W) Sholl analysis: multiple *t* tests using the two-stage linear step-up procedure of Benjamini, Krieger, and Yekutieli with *Q* = 1%; *P < 0.05; **P < 0.01; ***P < 0.001. Scale bars, 500 μm (A and D), 60 μm (B and E), and 10 μm (H to O).

In a first analysis, we quantified absolute numbers (per square centimeter) of mature cortical MADM-labeled green (GFP^+^) astrocytes in control-MADM (*Eed*^+/+^), *Eed*-MADM (*Eed*^−/−^), and cKO-*Eed*-MADM (*Eed*^−/−^) at P21. We observed significantly reduced numbers of *Eed*^−/−^ astrocytes in all cortical layers of *Eed*-MADM when compared to *Eed*^+/+^ astrocytes in control-MADM (Fig. 5, A to F, and fig. S7, E to H and K). The number of *Eed*^−/−^ astrocytes was even further diminished in cKO-*Eed*-MADM (fig. S7, I to K). The stronger decrease of astrocytes in cKO-*Eed*-MADM most probably reflects the combination of a cell-autonomous requirement of *Eed* for astrocyte production and the premature depletion of the progenitor pool during embryonic development (fig. S4).

To further address the role of PRC2 in gliogenic RGP lineage progression in more detail, we used mosaic *Eed*-MADM enabling the clear assessment of the cell-autonomous *Eed* function at the single-cell level. We performed time-course analysis (Fig. 5G, left) to determine whether reduced numbers of *Eed*^−/−^ astrocytes in *Eed*-MADM arise from an impairment in (i) neurogenic-to-gliogenic switch in RGPs, (ii) proliferation of astrocyte progenitors, or (iii) survival of astrocytes. We quantified GFP^+^ astrocytes in control-MADM and *Eed*-MADM mice at ages P4, P7, P10, P21, and 3 months (Fig. 5G, right). We detected comparable numbers of *Eed*^+/+^ and *Eed*^−/−^ nascent astrocytes at P4, indicating that the neurogenic-to-gliogenic switch in RGPs was not compromised upon loss of PRC2 activity. However, *Eed*^+/+^ control astrocytes underwent substantial expansion by proliferation in the first two postnatal weeks, indicated by increasing astrocyte numbers from P4 to P10. This observation was in agreement with recent findings (*47*), using lineage tracing at the clonal level to show that astrocytes mostly expand during the first postnatal week. In contrast, the proportion of *Eed*^−/−^ astrocytes remained rather constant from P4 onward and throughout the entire time course until 3 months (Fig. 5G, right). Thus, PRC2 cell-autonomously controls proliferation in astrocyte progenitors and/or promotes the survival rate of immature astrocytes.

Previous studies have shown that astrocytes undergo a morphological maturation phase from the first until the third postnatal week (*48*). We thus performed high-resolution imaging of individual green *Eed*^+/+^ and *Eed*^−/−^ astrocytes in time-course analysis (Fig. 5, H to O). We determined the total surface area (Fig. 5, P to S) and the extent of astrocyte process branching using Sholl analysis (Fig. 5, T to W). While the surface area and ramification of processes in *Eed*^+/+^ and *Eed*^−/−^ astrocytes were comparable at P4 (Fig. 5, P, Q, T, and U), we found that *Eed*^−/−^ astrocytes showed significant impairment in surface expansion from P7 and ramification of complex processes from P10 onward (Fig. 5, R, S, V, and W). We concluded that cell-autonomous PRC2 activity, besides controlling cortical astrocyte production, also fulfilled essential function in astrocyte maturation and branching.

### Transcriptomic signature of sparse immature *Eed*^−/−^ astrocytes correlates with their reduced proliferation potential

**Fig. 6.**
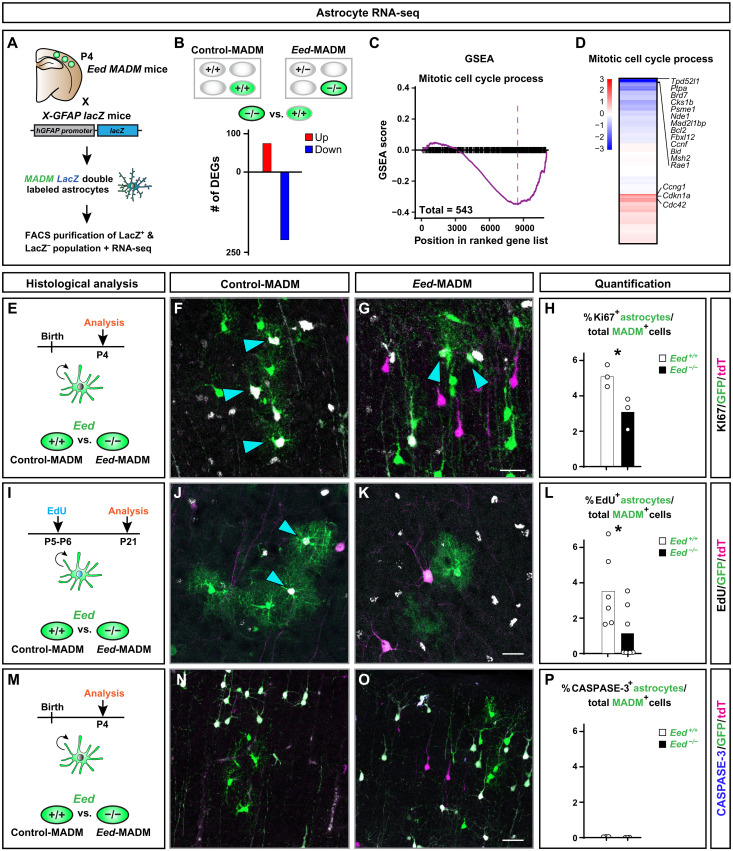
Cell-autonomous loss of *Eed* results in DEGs associated with mitotic cell cycle process, proliferation deficits, but no increase in apoptosis in developing cortical astrocytes. See also fig. S6. (**A**) Schematic overview of genetic strategy using the *X-GFAP lacZ* transgene in combination with MADM to isolate highly pure GFP^+^/lacZ^+^ astrocytes by FACS at P4 for RNA-seq. GFP^+^/lacZ^+^ astrocytes were sorted from control-MADM and *Eed*-MADM, respectively. (**B**) Number of DEGs (*P*_adj_ < 0.2, DESeq2) upon comparison of green *Eed*^−/−^ (*Eed*-MADM) and *Eed*^+/+^ (control-MADM) astrocytes. (**C**) Running score plot for the term mitotic cell cycle process determined by GSEA. (**D**) Heatmap representing gene expression of cell cycle genes in the term mitotic cell cycle process. Selected genes are indicated. (**E** to **H**) Proliferation analysis of cortical astrocytes at P4 using Ki67 staining. (E) Schematic overview of the experimental setup. (F to G) Representative images of GFP^+^ astrocytes in (F) control-MADM and (G) *Eed*-MADM. (H) Percentage of Ki67^+^ proliferating green *Eed*^+/+^ (white) and *Eed*^−/−^ (black) astrocytes. (**I** to **L**) Proliferation analysis of cortical astrocytes using EdU incorporation. (I) Schematic overview of the experimental setup with EdU injection at P5/P6 and analysis at P21. (J to K) Representative images of GFP^+^ astrocytes in (J) control-MADM and (K) *Eed*-MADM (L). Percentage of EdU^+^ proliferating green *Eed*^+/+^ (white) and *Eed*^−/−^ (black) astrocytes. (**M** to **P**) Cell death analysis of cortical astrocytes using caspase-3 stainings. (M) Schematic overview of the experimental setup with caspase-3 stainings in P4 cortex. (N and O) Representative images of GFP^+^ astrocytes in (N) control-MADM and (O) *Eed*-MADM. (P) Percentage of green *Eed*^+/+^ (white) and *Eed*^−/−^ (black) caspase-3^+^ astrocytes. Each individual data point represents one experimental animal. Statistics: Unpaired two-tailed *t* test with *P < 0.05. Data indicate mean ± SEM. Scale bars, 20 μm (F and G), 50 μm (J and K), and 25 μm (N and O).

To more comprehensively assess the phenotype of *Eed*^−/−^ astrocytes, we used a strategy that allowed us to isolate ultrapure astrocyte populations in MADM context (*37*, *38*). Briefly, we crossed *Eed*-MADM mice with transgenic *lacZ* reporter mice [*XGFAP-lacZ*; (*49*)], expressing *lacZ* under the control of the human *GFAP* promoter and thus highly specifically in cortical astrocytes. By using FACS, we purified MADM-labeled astrocytes (GFP^+^/lacZ^+^) and neurons (GFP^+^/lacZ^−^) from control-MADM and *Eed*-MADM mice at P4, when the numbers and morphology of green *Eed*^+/+^ and *Eed*^−/−^ astrocytes were comparable, and subjected respective samples to RNA-seq (Fig. 6A and data table S2). We confirmed the purity of isolated neuron and astrocyte populations by probing for the expression of specific cell identity markers. As expected, *Aqp4* and *Gfap* were highly enriched in astrocyte populations, while expression of *Lrrn3* and *Npm1* was much higher in neuron populations, independent of the *Eed* genotype (fig. S8, A and B). Next, we validated efficient *Eed* deletion in *Eed*^−/−^ FACS-isolated astrocytes (fig. S8C). To assess whether loss of PRC2 activity affects astrocyte identity, we analyzed the expression of a previously defined set of astrocyte-specific genes (*50*) in Eed^+/+^ and *Eed*^−/−^ astrocytes, respectively. We did not find consistent differences in marker gene expression for different astrocyte populations in *Eed*^+/+^ and *Eed*^−/−^ astrocytes. Thus, *Eed* deficiency seems to not alter astrocyte cell identity (fig. S8D). Upon comparison of the transcriptional profiles in *Eed*^+/+^ and *Eed*^−/−^ astrocytes, we identified 317 DEGs, of which most were down-regulated (Fig. 6B). Gene set enrichment analysis (GSEA) identified mitotic cell cycle process as a significantly enriched GO term, with most genes being down-regulated (Fig. 6, C and D). These results imply a cell-autonomous proliferation phenotype in *Eed*^−/−^ astrocytes.

To determine the biological significance of our RNA-seq analysis, we evaluated the levels of proliferation in *Eed*^+/+^ and *Eed*^−/−^ astrocytes in vivo. First, we assessed the percentage of Ki67^+^ green *Eed*^+/+^ and *Eed*^−/−^ astrocytes at P4 (Fig. 6E). We found a significant reduction in the proportion of Ki67^+^ *Eed*^−/−^ astrocytes when compared to *Eed*^+/+^ astrocytes (Fig. 6, F to H). Next, we performed EdU injections into pups at age P5 and P6 and analyzed the percentage of EdU^+^ green *Eed*^+/+^ or *Eed*^−/−^ astrocytes at P21 (Fig. 6I). We found reduced amounts of EdU^+^ *Eed*^−/−^ astrocytes when compared to *Eed*^+/+^ astrocytes (Fig. 6, J to L). To evaluate the level of apoptosis (potentially reducing numbers of *Eed*^−/−^ astrocytes), we stained for activated caspase-3 at P4 (Fig. 6M) but did not find any apoptotic figures in *Eed*^+/+^ and *Eed*^−/−^ astrocytes (Fig. 6, N to P). Together, our results demonstrate cell-autonomous requirement of *Eed* and thus PRC2 activity in cortical astrocyte production.

## DISCUSSION

In this study, we genetically dissected the cell type–specific cell-autonomous and global tissue-wide PRC2 functions in RGP lineage progression during neocortical development with unprecedented single-cell resolution. We used sparse and global *Eed* (essential component of PRC2) KO in combination with single-cell MADM-labeling paradigms. Against our prediction based on earlier work (*16*, *25*, *26*), we found that PRC2 activity is not cell-autonomously required in cortical neurogenesis but exerts critical functions rather at the global tissue-wide level. We demonstrated that the sparse loss of *Eed* in an individual RGP surrounded by phenotypically normal cells did not affect embryonic neurogenesis. In contrast, when *Eed* gene function was ablated in an all mutant environment, RGPs were compromised in their capacity to produce the correct number of cortical projection neurons. As a consequence, *Eed* cKO mice showed marked microcephaly. The distinct histological phenotypes of *Eed*^−/−^ mutant cells in *Eed*-MADM (normal cortex size) and cKO-*Eed*-MADM (microcephaly) were mirrored at the global transcriptome level. The mutant cellular environment in cKO-*Eed*-MADM developing cortex induced a unique gene expression pattern in *Eed*^−/−^ cells, which was highly distinct from the one in *Eed*^−/−^ cells upon sparse ablation.

**Fig. 7.**
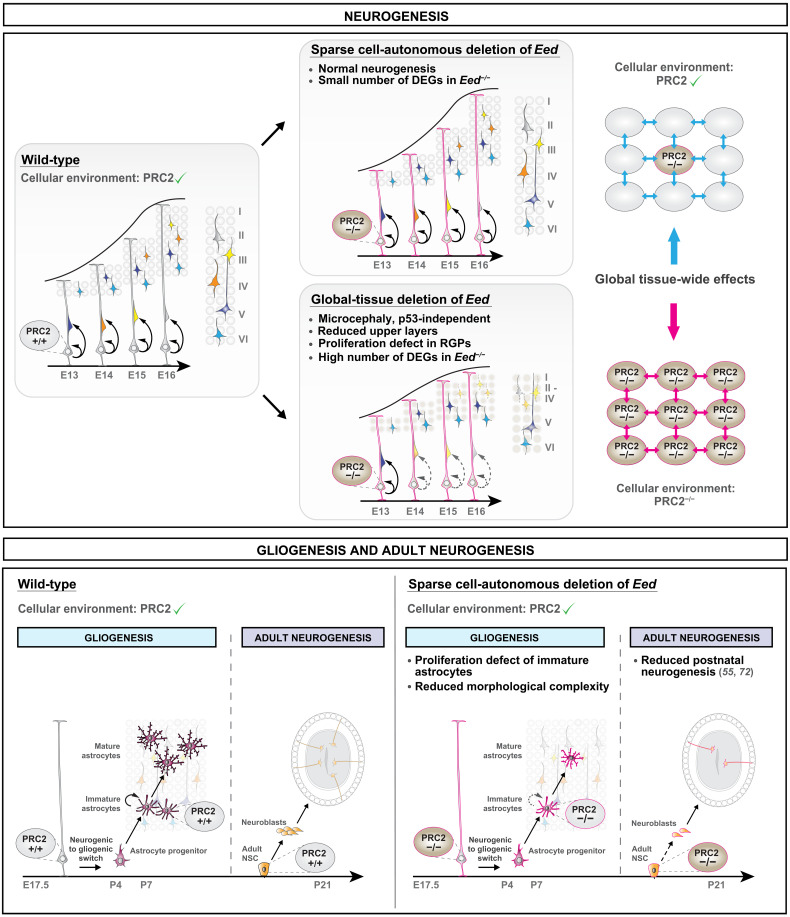
Distinct and sequential functions of PRC2 in neural stem cell lineage progression. Schematic model summarizing the findings of the study. PRC2 function is highly sensitive to the cellular environment during the neurogenic period. PRC2 is not cell-autonomously required to control cortical neurogenesis. Upon completion of RGP-mediated neurogenesis, PRC2 cell-autonomously regulates astrocyte production and maturation. Top: Individual *Eed*^−/−^ RGPs in *Eed*-MADM show a small number of deregulated genes (DEGs) and no deficits in cortical neurogenesis. In contrast, RGPs in cKO-*Eed*-MADM display a high number of DEGs and proliferation defects (*Trp53* independent) with diminished formation of upper layer neurons. Thus, global tissue-wide loss of *Eed* triggers non–cell-autonomous effects and leads to microcephaly. Bottom: *Eed*/PRC2 is cell-autonomously required in postnatal brain development for astrocyte production and maturation and adult neurogenesis. In line with previous studies (*55*, *72*), the sparse loss of *Eed* results in reduced postnatal neurogenesis with lower numbers of *Eed*^−/−^ granule cells in the olfactory bulb.

In contrast, H3K27me3, catalyzed by PRC2, was cell-autonomously required for cortical astrocyte production and maturation. Together, our MADM-based analysis revealed distinct and sequential *Eed*/PRC2 functions in RGP lineage progression (Fig. 7). Below, we discuss PRC2 requirement in cortical neurogenesis and astrocyte production in the context of individual cell-autonomous gene function and the interplay with tissue-wide genetic and cellular landscape.

### RGP proliferation deficits and microcephaly due to global PRC2 KO occurs independent of *Trp53* expression

The MADM system provided the unique opportunity to genetically dissect the level of cell autonomy of PRC2 function and the contribution of non–cell-autonomous tissue-wide genetic and cellular landscape in RGP lineage progression. Gene network analysis of DEGs specific for cKO-*Eed*-MADM has uncovered particular modules composed of functionally related genes. The core module encompasses proliferation-associated genes, correlating with diminished proliferation rates of RGPs in global PRC2 KO. Many primary microcephaly mutants show a strong mechanical impairment of cell division (defective assembly of the mitotic spindle or centromere attachment) (*41*–*45*, *51*, *52*) and a strong correlation between cell cycle mechanisms involved in DNA integrity, mitotic checkpoints, and prevention of DNA damage–induced apoptosis via the p53 pathway. Recent studies have unraveled a similar function for the chromatin remodelers Ino80 and REST. Both remodelers were shown to prevent accumulation of DNA damage and associated p53-dependent microcephaly in the forebrain (*51*, *52*).

On the basis of genetic epistasis experiments, we demonstrated that apoptosis in *Eed* cKO mice is p53 dependent; however, diminished RGP proliferation and associated microcephaly in *Eed* cKO mice could not be rescued by concomitant loss of *Trp53*. These findings point toward a *Trp53*-independent mechanism of progenitor loss and are insofar intriguing because other gene mutations affecting cell cycle progression trigger microcephaly in a *Trp53*-dependent manner (*41*–*45*, *51*, *52*). A recent study using transformed mouse embryonic fibroblasts revealed a PRC2-dependent mechanism to control cell proliferation independent of *Cdkn2a*-*Trp53-Rb1* (*53*). The authors provided evidence that Polycomb complexes actually localized at replication forks to direct progression and symmetry of DNA replication, thereby controlling cell proliferation independent of major cell cycle checkpoint genes and the p53 pathway. It is, however, not clear how cell cycle progression by Polycomb localization to replication forks is controlled at the individual cell level and which role the genetic landscape of the cellular environment plays in this process.

We can also not exclude the possibility that a separate individual deregulated gene other than a key component of the p53 signaling pathway may exist within the whole network, which may dominantly impair RGP proliferation in cKO-*Eed*-MADM. In principle, any single gene in the STRING network could be responsible for the microcephaly phenotype. As such, the phenotype-causing gene would stand out in the network based on the expectation to be a central node, connecting one module to DNA replication. Because we find heavy interconnection of the STRING network within and between modules, this scenario seems unlikely. On the basis of the network topology, we assume that multiple modules and multiple genes within each module act in concert to exert the proliferation phenotype observed in cKO-*Eed*-MADM mice.

### PRC2-dependent regulation of cortical neurogenesis and the relevance of global tissue-wide genetic and cellular environment

Given that the global mutant cellular landscape in cKO*-Eed*-MADM mice is the main driver of microcephaly, it is likely that the brain size alteration because of whole-tissue loss of PRC2 activity involves a complex aggregation or interplay of environmental cues and cell-intrinsic transcriptional responses on the global tissue level. Our data support a two-step process upon loss of PRC2 activity, leading to the terminal observed *Eed*^−/−^ phenotype in cKO-*Eed*-MADM mice. First, PRC2 deficiency triggers the up-regulation of a relatively small set of direct target genes, which in the second step induce deregulation of a much larger number of indirect and/or secondary target genes. The concerted net result of direct and indirect deregulated gene expression in cKO-*Eed*-MADM ultimately results in impaired RGP proliferation dynamics, elevated apoptosis, and global microcephaly. Two scenarios could explain the distinct phenotypic manifestation upon sparse and global KO of PRC2 activity. First, the heterozygous *Eed^+/−^* environment in *Eed*-MADM (sparse deletion) rescued RGPs from proliferation deficits by preventing exuberant deregulation of gene expression because of protective global tissue-wide effects. Second, the concerted loss of PRC2 activity in all cells in cKO-*Eed*-MADM triggers an exacerbated tissue-wide response that ultimately results in much increased ectopic expression of direct PRC2 target genes, thereby inducing secondary waves of ectopic transcription leading to the specific deregulation of genes controlling cell cycle and DNA replication. In other words, our interpretation of the underlying principle is pleiotropy caused by ectopic expression of PRC2 direct target genes, which encounters reciprocal feedback from surrounding mutant cells upon global loss of PRC2 activity. While the resulting secondary waves of gene expression changes are prevented by a normal cellular environment in *Eed*-MADM, the all-mutant environment in cKO-*Eed*-MADM cortices was able to respond to ectopic target gene expression and elicited the expression of further direct and downstream indirect target genes. We speculate that the exuberant gene expression changes in cKO-*Eed*-MADM were propagated through the tissue via environmental cues such as cell-cell interactions, secreted signaling molecules, and other extracellular components, which in turn depended on the altered gene expression profile of mutant cells. The systemic environment-dependent reduction of RGP proliferation in global *Eed* mutant cortex may be similar to the concept of a synthetic defect (i.e., a fitness defect upon whole-tissue loss of PRC2 activity in all cortical progenitor cells through simultaneous deregulation of multiple genes and/or gene modules). In conjunction with the network topology of cKO-specific DEGs, the synthetic defect strengthened our hypothesis of a multiple target model for the development of microcephaly in global tissue PRC2 mutants. Regardless of the precise mechanism, it becomes clear that the genetic and cellular environment is critical for RGP lineage progression and the generation of correctly sized cortex (Fig. 7, top).

### Cell-autonomous function of PRC2 in astrocyte development

A complex interplay of repressive mechanisms (e.g., DNA and histone methylation) and activating mechanisms (e.g., histone acetylation and chromatin remodeling) regulates RGP progression to gliogenesis and the fate choice between astrocyte and oligodendrocyte identity (*17*, *54*–*56*). PRC2 exhibits a highly dynamic spatiotemporal expression pattern during cortical development, with a significant loss of H3K27me3 levels in embryonic RGPs after E12.5 (*16*) and H3K27me3 regain in the early postnatal astrocyte lineage (fig. S7, A and B). On the basis of global tissue deletion studies, PRC2 was previously thought to play an essential role in regulating the neurogenic-to-gliogenic switch of RGPs (*25*, *26*). However, sparse cell-autonomous *Eed* deletion revealed that lineage progression of neurogenic RGPs to astrocyte intermediate progenitor cells (aIPCs) was unaffected, in terms of both numbers and molecular markers. These results underline that astrocyte specification following the neurogenic-to-gliogenic switch is not impaired by PRC2 deficiency at the single-cell level.

Following aIPC identity acquisition, cortical astrocytes are reported to scatter throughout all cortical layers while exhibiting a high proliferation rate during the first postnatal week (P0 to P7) (*47*). This dynamic dispersion and proliferation phase gradually progresses into a maturation phase (P7 to P21), where individual astrocytes increase in volume and process complexity (*47*). While many studies have addressed the function of epigenetic regulation during the onset of gliogenesis (*17*, *54*–*56*), the role of epigenetics in controlling proliferation and maturation of immature astrocytes is still largely unknown. Deposition of activating histone marks on astroglial genes precedes expression of these genes, indicating that transcription of key astroglial genes relies on precisely controlled activation of the respective loci (*57*). These findings imply that removal of repressive marks alone is not sufficient to induce transcription of key astroglial regulatory genes. On the basis of the single-cell resolution provided by our MADM approach, we were able to assess the consecutive steps of astrocyte development both quantitatively and qualitatively. Our results demonstrated that the loss of PRC2 had no effect on proper induction of astrocyte identity and differentiation, thus supporting the above rationale that PRC2-mediated mechanisms are dispensable for the transition of neurogenic-to-gliogenic RGPs. However, astrocyte maturation is accompanied by structural chromatin rearrangements consisting of both newly activated loci and silenced genes (*54*). Given that loss of PRC2 prevents de novo acquisition of H3K27me3-induced transcriptional silencing, ectopically expressed genes interfering with astrocyte maturation cannot be repressed. Thus, correct changes in chromatin accessibility mediated by PRC2 are essential to cell-autonomously safeguard correct astrocyte proliferation and maturation (Fig. 7, bottom).

### The implication of distinct and sequential PRC2 function in RGP lineage progression

Our findings add to several lines of evidence implying that sparse alteration of gene function at the individual cell level affects astrocyte production but is not cell-autonomously required for neurogenesis (*36*, *38*). However, certain genes only cause a significant disruption of faithful neurogenesis upon deletion at the global tissue level (*36*). The predominance of tissue-wide effects causing severe cortical malformations has also been recently reported for genes involved in migration or cell polarity (*58*), thus further underlining that global tissue or non–cell-autonomous systemic effects significantly shape the early steps of cortical development such as neuron production (*59*).

The discovery of distinct and sequential functions of PRC2 in lineage progression will have profound implications for our understanding of diseases arising from postzygotic mutations in individual dividing cells (i.e., somatic cell mosaicism). Somatic cell mosaicism can occur at any point throughout development and is associated with a number of disorders, including neurological diseases and tumorigenesis (*60*, *61*). In the future, it will be of great relevance to investigate the contribution of the cellular environment (i.e., the niche) to cell-autonomous or non–cell-autonomous phenotype manifestations upon acquisition of mutations altering the function of particular epigenetic mechanisms. Somatic glioma-promoting mutations altering the repressive mechanisms mediated by PRC2 were shown to affect H3K27me3 profiles [such as H3 or EZHIP mutations (*62*)]. Such mutations will be interesting candidates for the assessment of disease development originating from a single PRC2 mutant stem cell in a cell type– and environment-dependent manner.

## MATERIALS AND METHODS

A complete list of used reagents, materials, and software packages can be found in table S1.

### Mouse lines

All mouse colonies were maintained in accordance with protocols approved by institutional animal care and use committee, institutional ethics committee, and the preclinical core facility (PCF) at Institute of Science and Technology (IST) Austria. Experiments were performed under a license approved by the Austrian Federal Ministry of Science and Research in accordance with the Austrian and European Union animal law (license numbers: BMWF-66.018/0007-II/3b/2012 and BMWFW-66.018/0006-WF/V/3b/2017). The Austrian Federal Act on the Protection of Animals adheres to Institutional Animal Care and Use Committee (IACUC) guidelines. Mice with specific pathogen–free status according to the Federation of European Laboratory Animal Science Associations (FELASA) recommendations (*63*) were bred and maintained in experimental rodent facilities [room temperature (RT) 21° ± 1°C (mean ± SEM); relative humidity 40 to 55%; photoperiod 12L (12 hours light):12D (12 hours dark)]. Food (V1126, Ssniff Spezialitäten GmbH, Soest, Germany) and tap water were available ad libitum.

Mouse lines with MADM cassettes inserted in Chr. 7 (*32*), *Eed*-flox (*31*), *Trp53*-flox (*45*), *Trp53* null (*46*), *hGFAP-lacZ* (*49*), *Emx1*-Cre (*33*), and *Emx1*-Cre^ERT2^ (*35*) have been described previously. Calculation of recombination probability and recombination of *Eed-flox* allele onto chromosomes carrying the MADM cassettes was performed according to standard techniques. These are described in detail elsewhere (*34*) and are summarized in fig. S1. The MADM technique not only induces fluorescent labeling and thus unambiguous tracing of cells harboring mutations but also generates uniparental chromosome disomies (UPDs) (*38*). Cells harboring UPDs contain two copies of either the maternal chromosome (matUPD) or the paternal chromosome (patUPD). In our study, all mouse matings have been performed by crossing a MADM-7-GT female with a MADM-7-TG male. In this case, the offspring’s green cells will harbor patUPD and red cells will harbor matUPD (so-called forward mating). Mutant analysis can be separated from UPD effects (*29*, *38*) upon comparison of cells of the same UPD. Thus, contrasting the green cells with patUPD from control-MADM with those from *Eed*-MADM mice is the correct comparison to determine cell-autonomous *Eed* function in Eed-MADM cortices.

All MADM-based analyses were carried out in a mixed C57BL/6, CD1 genetic background, in male and female mice without sorting experimental cohorts according to sex. No sex-specific differences were observed under any experimental conditions or in any genotype. On the basis of genotype, experimental groups were randomly assigned. The age of experimental animals is indicated in the respective figures, figure legends, and source data files.

### Tissue isolation and immunostaining

From P4 onward, mice were deeply anesthetized by injection of a ketamine/xylazine/acepromazine solution (65, 13, and 2 mg/kg body weight), and unresponsiveness was confirmed through pinching the paw. The diaphragm of the mouse was opened from the abdominal side to expose the heart. Cardiac perfusion was performed with ice-cold PBS (phosphate-buffered saline) followed immediately by 4% PFA (paraformaldehyde) prepared in PB buffer. Brains were removed and further fixed in 4% PFA overnight to ensure complete fixation. Brains were cryopreserved with 30% sucrose (Sigma-Aldrich) solution in PBS for approximately 48 hours and were then embedded in Tissue-Tek O.C.T. (Sakura).

Pregnant females were sacrificed at the respective time points to obtain E12, E13, E14, E15, and E16 embryonic brain tissue. Embryonic and P0 brains were directly transferred into ice-cold 4% PFA. Cryopreservation was performed in 30% sucrose in PBS, and optimal cutting temperature (OCT) embedding was performed according to standard techniques. For embryonic time points and postnatal P0, 20- to 25-μm coronal frozen sections were directly mounted onto Superfrost glass slides (Thermo Fisher Scientific) and stored at −20°C until further usage.

For adult time points, 30- to 45-μm coronal frozen sections were collected in 24 multiwell dishes (Greiner Bio-One) and stored at −20°C in antifreeze solution [30% (v/v) ethylene glycol, 30% (v/v) glycerol, and 10% (v/v) 0.244 M PO_4_ buffer] until use. Embryonic sections were removed from −20°C and allowed to acquire RT for 15 min, followed by three wash steps (5 min) with PBS. Tissue sections were blocked for 30 min in a blocking buffer [containing 5% horse serum (HS) (Thermo Fisher Scientific) and 1% Triton X-100 in PBS]. Primary antibodies were diluted in staining buffer (5% HS and 0.5% Triton X-100) and incubated overnight at 4°C. Sections were washed three times for 5 min with PBS and incubated with corresponding secondary antibody diluted in staining buffer for 2 hours at RT. Sections were washed three times with PBS. Adult brain sections were transferred to a 24-well plate filled with PBS and stained as floating sections following the abovementioned steps. After the washes following the secondary antibody staining, sections were mounted onto Superfrost glass slides (Thermo Fisher Scientific) and allowed to dry. Nuclear staining of glass-mounted brain sections was performed by 10-min incubation with PBS containing 2.5% DAPI (4′,6-diamidino-2-phenylindole) (Thermo Fisher Scientific). Sections were embedded in mounting medium containing 1,4-diazabicyclooctane (Roth) and Mowiol 4-88 (Roth) and stored at 4°C until imaging.

Primary antibodies used are as follows: chicken anti-GFP (AVES; dilution 1:400), goat anti-mCherry (SICGEN; dilution 1:400), rabbit anti-CTIP (Abcam; dilution 1:400), goat anti-CUX1 (Santa Cruz Biotechnology; dilution 1:100), rabbit anti–caspase-3 (Cell Signaling Technology; dilution 1:500), rabbit anti-H3K27me3 (Diagenode; dilution 1:1000), goat anti-SOX2 (Santa Cruz Biotechnology; dilution 1:500), rat anti-TBR2 (Thermo Fisher Scientific; dilution 1:500), and rabbit anti-NEUROD2 (Abcam; dilution 1:200). Secondary antibodies used are as follows: donkey anti–chicken–fluorescein isothiocyanate (FITC) secondary antibody (Invitrogen; dilution 1:500), donkey anti-goat Alexa Fluor 568 secondary antibody (Molecular Probes; dilution 1:1000), donkey anti-goat Alexa Fluor 647 secondary antibody (Molecular Probes; dilution 1:1000), donkey anti-rabbit Alexa Fluor 647 secondary antibody (Molecular Probes; dilution 1:1000), donkey anti-rat Alexa Fluor 568 secondary antibody (Molecular Probes; dilution 1:1000), and donkey anti-goat Alexa Fluor 488 secondary antibody (Molecular Probes; dilution 1:1000).

### MADM clonal analysis

MADM clone analysis was performed as previously described (*28*). Briefly, timed pregnant females were injected intraperitoneally with TM (Sigma-Aldrich) dissolved in corn oil (Sigma-Aldrich) at E11 or E12 at a dose of 2 to 3 mg per pregnant dam. Live embryos were recovered at E18 and E19 through cesarean section, fostered, and raised for further analysis at P21. For embryonic time point analysis, cesarean section and analysis were performed at either E13 or E16. Brains containing MADM clones were isolated, and tissue sections were processed as described above.

### EdU labeling experiments

Cell cycle experiments were based on the use of a Click-iT Alexa Fluor 647 imaging kit (Thermo Fisher Scientific, C10340). Reagents were reconstituted according to the user manual. Pregnant females were injected with EdU (1 mg/ml EdU stock solution; 50 μl/10 g mouse) at E14.5. Embryos were collected 24 hours after EdU injection. Tissue was fixed in 4% PFA, and immunohistochemistry was performed as described above, except that the Click-iT imaging kit was used (according to the instruction manual) to visualize the EdU signal before performing the DAPI staining. P5/P6 pups were injected with EdU [EdU stock solution (1 mg/ml); 30 μl per mouse]. Mice were perfused at P21 using 4% PFA. Immunohistochemistry was performed as described above, except that the Click-iT imaging kit was used (according to the instruction manual) to visualize the EdU signal before performing the DAPI staining.

### Imaging and quantification

Sections were imaged using an inverted LSM800 confocal microscope (Zeiss) and processed using Zeiss Zen Blue software. Confocal images were analyzed in Photoshop software (Adobe). For population analysis, a minimum of six representative sections harboring the somatosensory cortex were imaged per individual brain. For clonal analysis, all consecutive sections harboring one clone were imaged.

### Preparation of single-cell suspension and FACS

For embryonic tissue, pregnant females were sacrificed by cervical dislocation and E12/E13/E16 embryos were collected. Embryos were sacrificed by decapitation. For postnatal tissue, pups were isolated from their mothers and were sacrificed by decapitation (P0) or cervical dislocation (P4 onward). Next, the neocortex area was dissected. For MADM cortical bulk sorting, pools of cortices derived from two to three individual mice were used to generate one biological sample. For astrocyte sorting, individual animals were used to generate biological replicates. Single-cell suspensions were prepared by using papain containing l-cysteine and EDTA (vial 2, Worthington), deoxyribonuclease (DNase) I (vial 3, Worthington), ovomucoid protease inhibitor (vial 4, Worthington), Earle’s Balanced Salts (EBSS) (Thermo Fisher Scientific), Dulbecco’s modified Eagle’s medium (DMEM)/F12 (Thermo Fisher Scientific), fetal bovine serum (FBS), and horse serum (HS). All vials from the Worthington kit were reconstituted according to the manufacturer’s instructions using EBSS. The dissected brain area was directly placed into papain-DNase solution [papain (20 U/ml) and 1000 U of DNase]. Enzymatic digestion was performed for 30 min at 37°C in a shaking water bath. Next, solution 2 [EBSS containing 0.67 mg of ovomucoid protease inhibitor and DNase I (166.7 U/ml)] was added, and the whole suspension was thoroughly mixed and centrifuged for 5 min at 1000 rpm at RT. Supernatant was removed, and cell pellet was resuspended in solution 2. Trituration with p1000 pipette was performed to mechanically dissolve any remaining tissue parts. DMEM/F12 was added to the cell suspension as a washing solution, followed by a centrifugation step of 5 min with 1500 rpm at RT. For GFP^+^ bulk sorting, cells were resuspended in medium (DMEM/F12 containing 10% FBS and 10% HS) and kept on ice until sorting.

For GFP^+^ astrocyte sorting, cells were incubated for 25 min at 37°C in 100 μl of LacZ staining reagent (Abcam, diluted 1:50). Reaction was stopped by adding 100 μl of DMEM/F12 containing 10% FBS and 10% HS. Samples were centrifuged for 5 min with 1500 rpm at RT and resuspended in DMEM/F12 containing 10% FBS and 10% HS. Samples were kept in the dark at RT until sorting was started. To ensure specificity of LacZ staining, a negative sample was always processed and sorting gates were adjusted accordingly. See further details at (*37*).

Right before sorting, cell suspension was filtered using a 40-μm cell strainer. FACS was performed on BD FACSAria III using 100 nozzle and keeping sample and collection devices (0.8-ml polymerase chain reaction tubes) at 4°C. Duplet exclusion was performed to ensure sorting of true single cells. Next, the maximum number of GFP^+^ cells from one individual sample was sorted. We collected the GFP^+^ cells to allow assessment of transcriptional profiles of *Eed*^+/+^ cells from control-MADM, green *Eed*^−/−^ cells from *Eed*-MADM, and green *Eed*^−/−^ cells from cKO-*Eed*-MADM mice. The MADM technique not only induces fluorescent labeling and thus unambiguous tracing of cells harboring mutations but also generates UPDs (*38*). Cells harboring UPDs contain two copies of either the matUPD or the patUPD. In our study, all mouse matings have been performed by crossing a MADM-7-GT female with a MADM-7-TG male. In this case, the offspring’s green cells will harbor patUPD and red cells will harbor matUPD (so-called forward mating). The UPDs induced by the MADM technique may influence gene expression profiles. To rigorously identify DEGs based on PRC2 deficiency and independent of UPD effects, we only assessed cells of the same UPD (i.e., GFP^+^ cells from forward mating).

In our study, cells of correct UPD are the green cells. Astrocytes and E12.5 GFP^+^ cells were sorted into 4 μl of lysis buffer [0.2% Triton X-100 and ribonuclease (RNase) inhibitor (2 U/μl) (Clontech)], while E13.5, E16.5, and P0 GFP^+^ bulk samples were sorted into 50 μl of RNA extraction buffer [10 mM EDTA (pH 8.0), 30 mM EDTA (pH 8.0), 1% SDS, and proteinase K (200 μm/μl) in nuclease-free water]. Immediately after sorting into 4 μl of lysis buffer was completed, samples were transferred into a 96-well plate (Bio-Rad) that was kept on dry ice. Once the plate was full, it was sealed with aluminum seal and kept at −80°C until further processing.

### RNA extraction and complementary DNA library preparation of MADM samples for RNA-seq

After cell sorting of E13/E16 and P0 cells, samples were incubated for 30 min at 37°C and stored at −80°C until further usage. After thawing samples on ice, their total volume was adjusted to 250 μl using RNase-free H_2_O (Thermo Fisher Scientific), followed by the addition of 750 μl of TRIzol LS (Thermo Fisher Scientific) and inverting five times. After incubating for 5 min at RT, the entire solution was transferred into a MaXtract tube (QIAGEN). Chloroform (200 μl) (Sigma-Aldrich) was added. After three times 5-s vortexing and 2-min incubation at RT, samples were centrifuged for 2 min with 12,000 rpm at 18°C. Supernatant was transferred to a new tube, and isopropanol (Sigma-Aldrich) was added in a 1:1 ratio. For better visibility of the RNA pellet, 1 μl of GlycoBlue (Thermo Fisher Scientific) was added and the entire solution was mixed by vortexing 3 × 5 s. Samples were incubated at −20°C for three nights to precipitate RNA. Then, samples were centrifuged for 20 min with 14,000 rpm at 4°C. Supernatant was removed, and RNA pellet was washed with 70% ethanol, followed by a 5-min centrifugation step (14,000 rpm at 4°C). RNA pellet was resuspended in 12.5 μl of RNase-free H_2_O. RNA quality was analyzed using a Bioanalyzer RNA 6000 Pico kit (Agilent) following the manufacturer’s instructions. RNA samples were pipetted into a 96-well plate, sealed with aluminum seal, and stored at −80°C until further use. Sequencing libraries were prepared following the Smart-Seq v2 protocol (*64*) using custom reagents (VBCF GmbH), and libraries from a 96-well plate were pooled, diluted, and sequenced on HiSeq 2500 (Illumina) using v4 chemistry or NextSeq550 (Illumina).

### Quantification and statistical analysis

#### Analysis of MADM-labeled brains

Sections were imaged using an inverted LSM800 confocal microscope (Zeiss) and processed using Zeiss Zen Blue software. Confocal images were imported into Photoshop software (Adobe), and MADM-labeled cells were manually counted in the somatosensory cortex based on morphology or respective marker expression as described previously (*2*, *36*) (Figs. 1 , 2, and 4 to 6). Cortical areas were identified by using the Allen Brain Atlas (http://mouse.brain-map.org/static/atlas). To determine cortical thickness, solely DAPI images were used. Images were opened in Zen Blue software, and measurements were performed using the “line”-tool of this software. Three different measurements in the somatosensory cortex were done per image and combined to one value by averaging the three measured values (Figs. 1 and 5). For cell quantification and cortical thickness measurements, a minimum of six sections were analyzed per individual brain. A minimum of three different individuals were analyzed per genotype. Data visualization was performed in GraphPad Prism 7.0. Significance was determined using unpaired *t* test or one-way or two-way analysis of variance (ANOVA) with multiple comparisons tests in GraphPad Prism 7.0. A detailed list of sample size, quantified sections, cells, and statistics is provided in data table S3. All source data of quantifications are provided in data table S4.

For the assessment of the green/red neuron ratio, the absolute number of green and red neurons in control-MADM, *Eed*-MADM, and cKO-*Eed*-MADM cortices was quantified. For the assessment of astrocyte numbers, GFP^+^ astrocytes in control-MADM, *Eed*-MADM, and cKO-*Eed*-MADM mice were quantified and normalized to cortex area.

For clonal analysis (Fig. 2), two-dimensional (2D) clone reconstruction was performed by using a custom script in ImageJ (*28*). For a detailed protocol, please refer to (*28*). For astrocyte morphological analysis, green *Eed*^+/+^ astrocytes from control-MADM and green *Eed*^−/−^ astrocytes from *Eed*-MADM mice were compared to each other.

#### Astrocyte morphology analysis

Detailed analysis of astrocyte morphology was done as described previously (*36*) with some modifications. Lower layer astrocytes that expressed GFP^+^ were imaged with a 63× oil objective and overlapping *z*-sections. 3D reconstruction and analysis were done using the Filament tracer algorithm of the IMARIS software. The total cell volume of astrocytes was assessed from the 3D structure. Sholl analysis was performed to measure astrocyte branching complexity.

#### Processing and analysis of bulk RNA-seq data from embryonic time course

Read processing, alignment, and annotations were described previously (*65*). STAR (*66*) alignment parameters: --outFilterMultimapNmax 1, --outSAMstrandField intronMotif, --outFilterIntronMotifs RemoveNoncanonical, and quantMode GeneCounts. Downstream analyses were performed in R (v3.6.1, v4.1.2). Read counts of the deleted region in the *Eed* gene (chr7:89969506-89972357, mm10) were calculated using bedtools (*67*) intersect with the -split option on the aligned bam file produced by STAR.

We analyzed 68 samples of GFP^+^ cells and removed 16 samples with a low percentage of uniquely aligned reads (<60%) and low absolute number of uniquely aligned reads (<1 million) because of low correlation with biological replicates or because of high level of read counts in *Eed* deleted region indicating inefficient recombination.

Figure S3B: Read counts in *Eed* deleted regions were calculated as reads per million total reads and plotted.

Figure 3E and fig. S3C: Statistics on differential expression between all pairs of genotypes were calculated with DESeq2 (v1.26) (*68*) using contrasts for each developmental time point separately. To reduce noise, only genes with an average read coverage of >1 (E12.5) or >10 (E13.5, E16.5, and P0) were used in the analyses. We used an adjusted *P* value (*P*_adj_) cutoff of 0.05 for DEGs and a log_2_ fold change of >0 (up-regulation) or <0 (down-regulation) for analyses.

Figure S3C: Score heatmap: All DEGs from all developmental time points were analyzed. For each gene, the log_10_ of the uncorrected *P* value, from the indicated comparison, was calculated and corrected to be positive for log_2_ fold change > 0 and negative for log_2_ fold change < 0. This score was cut at +5/−5 for better visualization. Heatmap was drawn with pheatmap package (v1.0.12).

Figure 3G and fig. S5B: To determine the coverage of genes with H3K27me3, we used published data from (*39*). We downloaded data set 1 (embj201796764-sup-0002-datasetev1.xlsx) and used columns 9 to 11 (aRG-P, aRG-N, and bRG) for further analyses. Genes with duplicated symbol IDs were removed. Direct target genes were defined as up-regulated in the respective DEG analysis (log_2_ fold change > 0) and reported to be marked in one or more of the abovementioned cell types by H3K27me3. Indirect target genes were defined as either down-regulated or not marked by H3K27me3. Note that for this analysis, we used DEGs (*P*_adj_ < 0.05) from *Eed*-MADM/control-MADM and cKO-*Eed*-MADM/control-MADM analyses that were also informative in (*39*). Because the overlap of genes between both data sets is not complete, the sum of genes in Fig. 3G is smaller than in Fig. 3F.

Figure 3I and fig. S5 (C to F): This analysis was performed with R v4.1.2. To identify protein-protein interactions, we used the STRING database via the R package STRINGdb (v2.6.5). A STRINGdb object was initialized with STRINGdb$new and parameters: version = “11.0”, species = 10090, score_threshold = 300. We mapped the gene symbols of the 457 cKO-*Eed*-MADM–specific DEGs and 88 DEGs resulting from the combination of *Eed*-MADM–specific and common (shared between cKO-*Eed*-MADM and *Eed*-MADM) DEGs to STRING IDs using the function map. Ninety-five percent of genes could be mapped in this way. To obtain an overview of the STRING networks and to identify the significance of the retrieved protein interaction networks, we used the function plot_network with the results shown in fig. S5C. Next, we identified protein-protein interactions using the get_interactions function (STRINGdb). We created an igraph (v1.2.5) object using graph.data.frame with directed = F parameter, removed multiple edges, and identified connected subnetworks using the function clusters. We extracted the largest connected network (400 proteins for cKO-*Eed*-MADM, 25 for Eed-MADM and common). Because only the cKO-*Eed*-MADM analysis revealed a significantly connected network, we focused the following analyses on the largest connected network from cKO-*Eed*-MADM. To visualize the network, we exported the igraph object to Cytoscape (v3.7.2/v3.8.1) (*69*) using createNetworkFromIgraph function from RCy3 package (v2.14.2) (*70*). We identified clusters of genes using get_clusters function (STRINGdb) with standard parameters. This analysis grouped the 400 genes in 10 clusters. Three of these clusters (clusters 8, 9, and 10 in data table S1) were extremely small with 3, 3, and 2 genes, respectively, and were thus removed from further analysis. Genes in the remaining clusters were analyzed using enrichGO (clusterProfiler package v4.2.2) (*71*) with OrgDb = org.Mm.eg.db (v3.14.0), ont = “all,” readable = T, pool = T parameters. A score was calculated as the negative log_10_ of the *P*_adj_ value and used to plot the top three enriched GO terms in fig. S5F.

Figure 5F: Edge thickness correlates to number protein-protein interactions between clusters.

#### Processing and analysis of bulk RNA-seq data from purified astrocytes

We analyzed 16 samples and removed 5 samples based on position on principle component analysis (PCA) plot or low correlation between biological replicates. All normalized gene expression values were identified using DESeq2’s counts function with normalize = T parameter. Heatmaps were drawn with pheatmap.

Figure S8 (A and B): For astrocyte marker genes, the median normalized gene expression for neuron samples was set to 1, and the expression of astrocyte samples was plotted relative to that value. For neuron marker genes, the median normalized expression for astrocyte samples was set to 1, and the expression of neuron samples was plotted relative to that value.

Figure S8C: *Eed* deletion counts were identified as described above, and normalized expression values are shown.

Figure S8D: Normalized expression values of astrocyte markers identified by (*50*) were averaged over all biological replicates. The highest expressing sample was set to 1, and expression of the respective other sample is shown relative to that sample.

Figure 6B: Statistics on differential expression between genotypes were calculated with DESeq2 (v1.26) (*68*). We used a *P*_adj_ cutoff of 0.2 for DEG and a log_2_ fold change of >0 (up-regulation) or <0 (down-regulation) for analyses.

Figure 6C: For GSEA, a score was calculated as the log_10_ uncorrected *P* value and corrected to be positive for log_2_ fold change > 0 and negative for log_2_ fold change < 0. DEGs were sorted on the basis of this score and analyzed using gseGO from the clusterProfiler package (v3.14.3) (*71*) using ont = “BP,” OrgDb = org.Mm.eg.db (v3.10.0), pvalueCutoff = 0.9, minGSSize = 500, and maxGSSize = 1000 parameters. The term mitotic cell cycle process was the top enriched term with a *P* value of 0.011. Running scores were plotted using gseaplot from the clusterProfiler package.

Figure 6D: Score heatmap is as described above with genes from GO term “mitotic cell cycle progress” values cut at +3/−3.

## Notes

View/request a protocol for this paper from *Bio-protocol*.
