# Supplementary material for: Tissue-wide genetic and cellular landscape shapes the execution of sequential PRC2 functions in neural stem cell lineage progression
Source: Sci Adv. 2022 Nov 2;8(44):eabq1263. doi: 10.1126/sciadv.abq1263 (PMC9629739; doi:10.1126/sciadv.abq1263)
Supplement: Supplementary file 1 — Figs. S1 to S8 Table S1 [file sciadv.abq1263_sm.pdf]

Science Advances
AAAS

Supplementary Materials for

Tissue-wide genetic and cellular landscape shapes the execution of sequential PRC2 functions in neural stem cell lineage progression

Nicole Amberg *et al.*

Corresponding author: Simon Hippenmeyer, simon.hippenmeyer@ist.ac.at

Sci. Adv. **8**, eabq1263 (2022)
DOI: 10.1126/sciadv.abq1263

The PDF file includes:

Figs. S1 to S8
Table S1
Legends for data tables S1 to S4

Other Supplementary Material for this manuscript includes the following:

Data tables S1 to S4

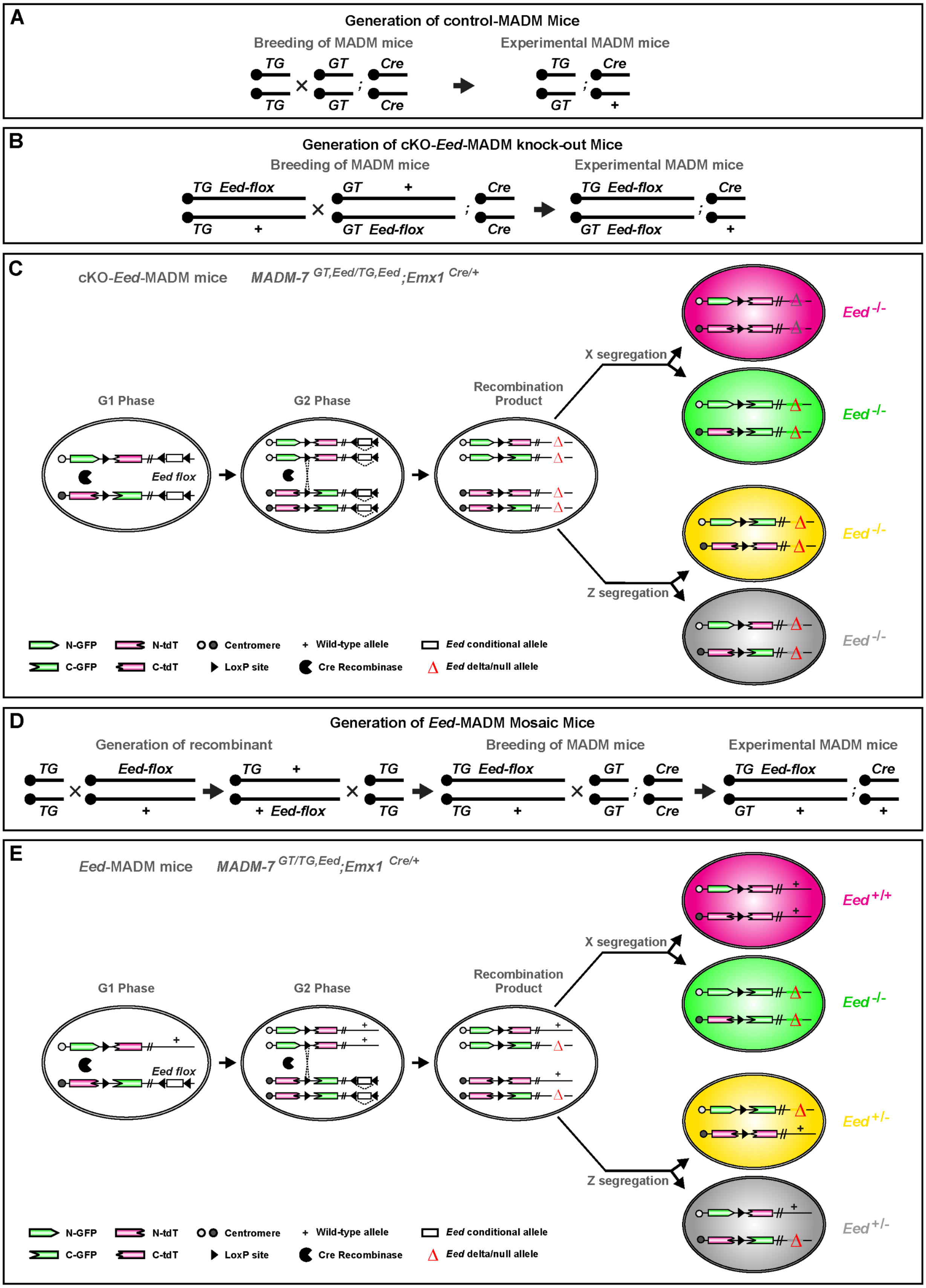
A
Generation of control-MADM Mice
Breeding of MADM mice
Experimental MADM mice
TG TG × GT GT ; Cre Cre
TG GT ; Cre +
B
Generation of cKO-Eed-MADM knock-out Mice
Breeding of MADM mice
Experimental MADM mice
TG Eed-flox TG + × GT + GT Eed-flox ; Cre Cre
TG Eed-flox GT Eed-flox ; Cre +
C
cKO-Eed-MADM mice MADM-7 GT,Eed/TG,Eed;Emx1 Cre/+
G1 Phase
G2 Phase
Recombination Product
Eed flox
X segregation
Z segregation
Eed -/-
Eed -/-
Eed -/-
Eed -/-
N-GFP
C-GFP
N-tdT
C-tdT
Centromere
LoxP site
Wild-type allele
Cre Recombinase
Eed conditional allele
Eed delta/null allele
D
Generation of Eed-MADM Mosaic Mice
Generation of recombinant
Breeding of MADM mice
Experimental MADM mice
TG TG × Eed-flox + → TG + + Eed-flox × TG TG → TG Eed-flox TG + × GT GT ; Cre Cre → TG Eed-flox GT + ; Cre +
E
Eed-MADM mice MADM-7 GT/TG,Eed;Emx1 Cre/+
G1 Phase
G2 Phase
Recombination Product
Eed flox
X segregation
Z segregation
Eed +/+
Eed -/-
Eed +/-
Eed +/-
N-GFP
C-GFP
N-tdT
C-tdT
Centromere
LoxP site
Wild-type allele
Cre Recombinase
Eed conditional allele
Eed delta/null allele

Figure S1. Related to Figure 1. MADM-based experimental paradigms for the generation of *Eed* mutant cells with single cell resolution.

(A) Breeding strategy for the generation of control-MADM mice.

(B) Breeding strategy for the generation of cKO-*Eed*-MADM mice.

(C) MADM scheme for *Eed* conditional knockout (cKO). The *Eed* conditional allele was introduced distal to both, the GT-MADM and the TG-MADM cassettes, via meiotic recombination (*29, 34*). Following Cre-mediated interchromosomal recombination and mitosis, sparsely labelled GFP^+ (green), tdT^+ (red) and GFP^+ tdT^+ (yellow) homozygous *Eed* mutant cells will be generated in an unlabeled homozygous *Eed* mutant environment.

(D) Breeding strategy for the generation of mosaic *Eed*-MADM mice.

(E) MADM scheme for sparse mosaic *Eed* knockout. The *Eed* conditional allele was introduced distal to the TG-MADM cassette via meiotic recombination (*29, 34*). Upon G2-X event one GFP^+ homozygous *Eed* mutant cell and one tdT^+ homozygous wild-type cell will be generated in an unlabeled heterozygous environment. G2-Z segregation results in one unlabeled and one yellow heterozygous cell (*29, 34*).

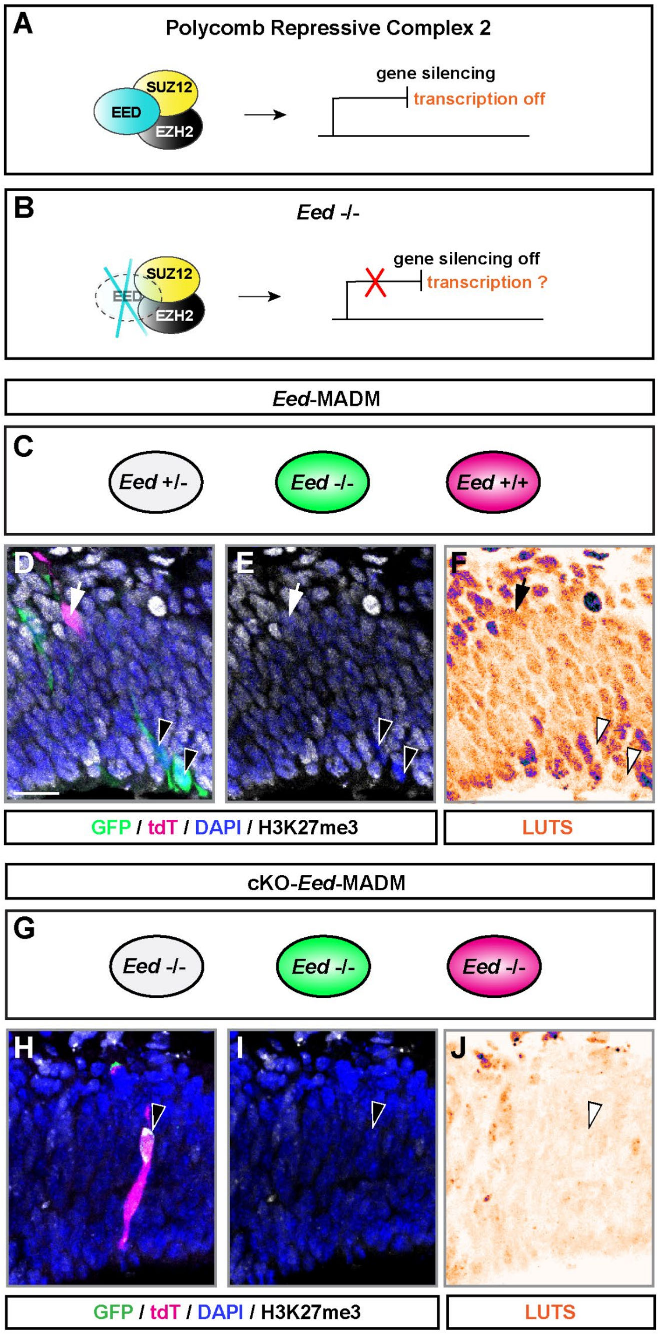

Figure S2. Related to Figure 1. Validation of PRC2 inactivation in experimental MADM paradigms.

(A) Schematic overview of PRC2 core components and function.

(B) Schematic overview of genetic deletion of PRC2 activity by using floxed alleles of *Eed*.

(C) Depiction of cellular genotypes in *Eed*-MADM (sparse *Eed* deletion).

(D-F) H3K27me3 staining in *Eed*-MADM cortex at E12.5, confirming absence of H3K27me3 and thus PRC2 activity specifically in green *Eed*$^{-/-}$ cells. (E) H3K27me3 and DAPI only. (F) LUTS display of H3K27me3 staining intensity. Arrowheads point on individual green *Eed*$^{-/-}$ cells which show absence of H3K27me3 mark, while full arrow points on a red *Eed*$^{+/+}$ cell which, like the unlabeled cellular environment, is positive for H3K27me3.

(G) Depiction of cellular genotypes in cKO-*Eed*-MADM cortex (global tissue-wide *Eed* KO).

(H-J) H3K27me3 staining in cKO-*Eed*-MADM cortex at E12.5 confirming absence of H3K27me3 and thus PRC2 activity in all neural cells. (I) H3K27me3 and DAPI only. (J) LUTS display of H3K27me3 staining intensity. Arrowhead points on an individual yellow *Eed*$^{-/-}$ cell which shows absence of the H3K27me3 mark, like the unlabeled cellular environment.

Scale bar in (D-F, H-J): 20µm.

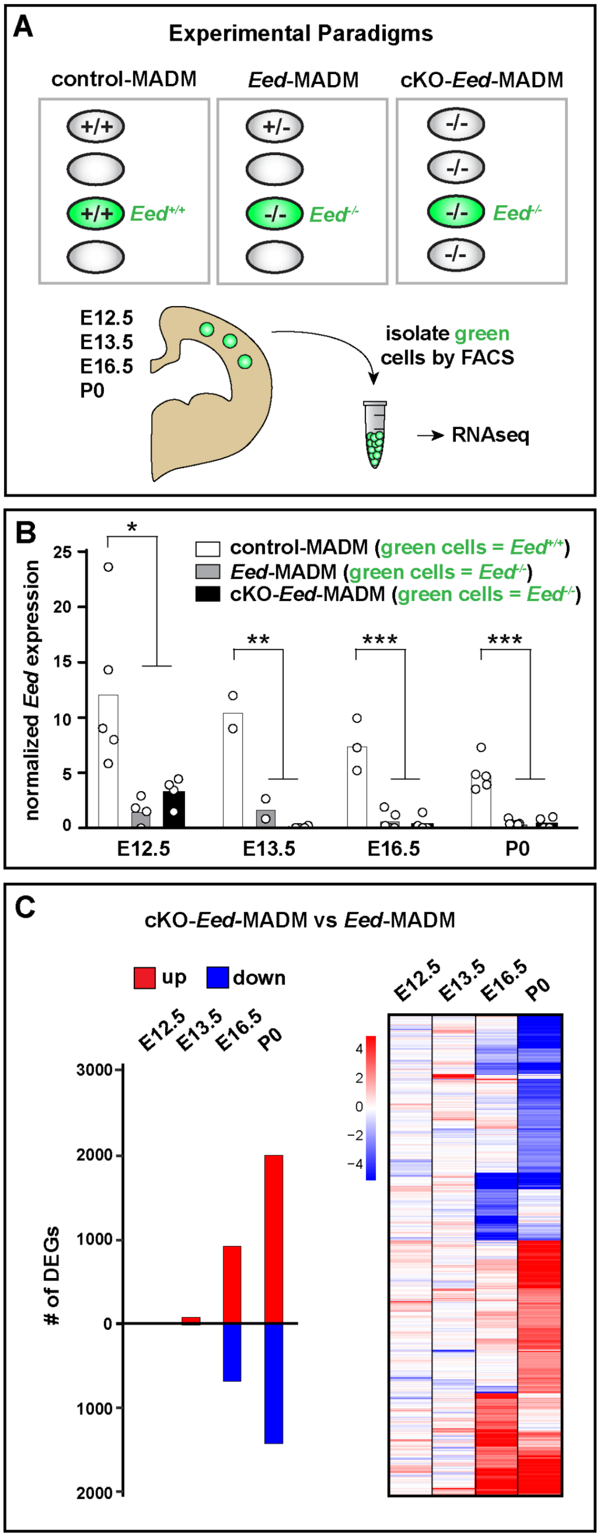

A
Experimental Paradigms
control-MADM
Eed-MADM
cKO-Eed-MADM
+/+
+/-
-/-
Eed+/+
Eed-/-
E12.5
E13.5
E16.5
P0
isolate green cells by FACS
RNAseq
B
normalized Eed expression
control-MADM (green cells = Eed+/+)
Eed-MADM (green cells = Eed-/-)
cKO-Eed-MADM (green cells = Eed-/-)
E12.5
E13.5
E16.5
P0
C
cKO-Eed-MADM vs Eed-MADM
up
down
# of DEGs
E12.5
E13.5
E16.5
P0

Figure S3. Related to Figure 3. Gene expression analysis in MADM-labeled *Eed*$^{-/-}$ cells upon sparse and global tissue-wide *Eed* deletion.

(A) Schematic overview of experimental MADM paradigms and genotype of GFP^{+} cells in control-MADM; *Eed*-MADM; and cKO-*Eed*-MADM.

(B) Normalized *Eed* expression at E12.5, E13.5, E16.5 and P0 in purified green cells from (white) control-MADM, (grey) *Eed*-MADM and (black) cKO-*Eed*-MADM.

(C) Graphical representation of the number of DEGs upon comparison of *Eed*$^{-/-}$ mutant cells in *Eed*-MADM and cKO-*Eed*-MADM at 12.5, E13.5, E16.5 and P0 (left). Score heat map of DEGs from 12.5, E13.5, E16.5 and P0 upon comparison of *Eed*$^{-/-}$ mutant cells in *Eed*-MADM and cKO-*Eed*-MADM (right).

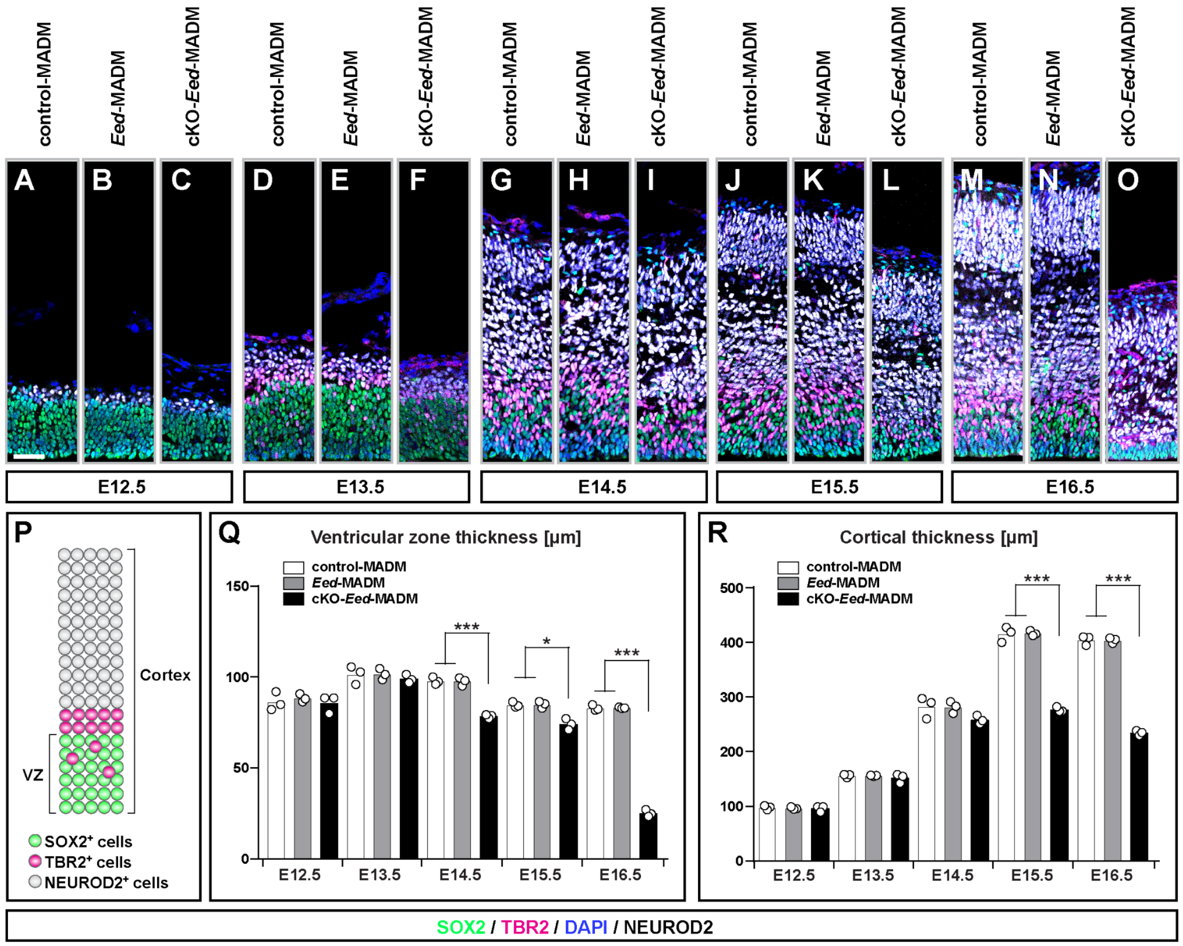

Figure S4. Related to Figure 3. Embryonic time course analysis of the progenitor pool in vivo.

(**A-O**) Immunofluorescence staining of RGPs (SOX2, green), intermediate progenitors (TBR2, red), and immature neurons (NEUROD2, white) in control-MADM, *Eed*-MADM and cKO-*Eed*-MADM cortices at E12.5 (A-C), E13.5 (D-F), E14.5 (G-I), E15.5 (J-L) and E16.5 (M-O).

(**P**) Schematic illustration of determination of ventricular zone (VZ) and complete cortex from SOX2 TBR2 NEUROD2 stainings shown in A-O.

(**Q-R**) Quantification of ventricular zone thickness (Q) and complete cortical thickness (R) from mice of indicated genotypes and ages. Statistics: one-way ANOVA with Turkey's multiple comparisons; * = $p < 0.05$; ** $p < 0.01$; *** $p < 0.001$. Data show mean ± SEM.

Each individual data point represents one experimental animal.

Statistics: one-way ANOVA with multiple comparisons; * $p<0.05$; ** $p<0.01$; *** $p< 0.001$.

Scale bar: 20µm.

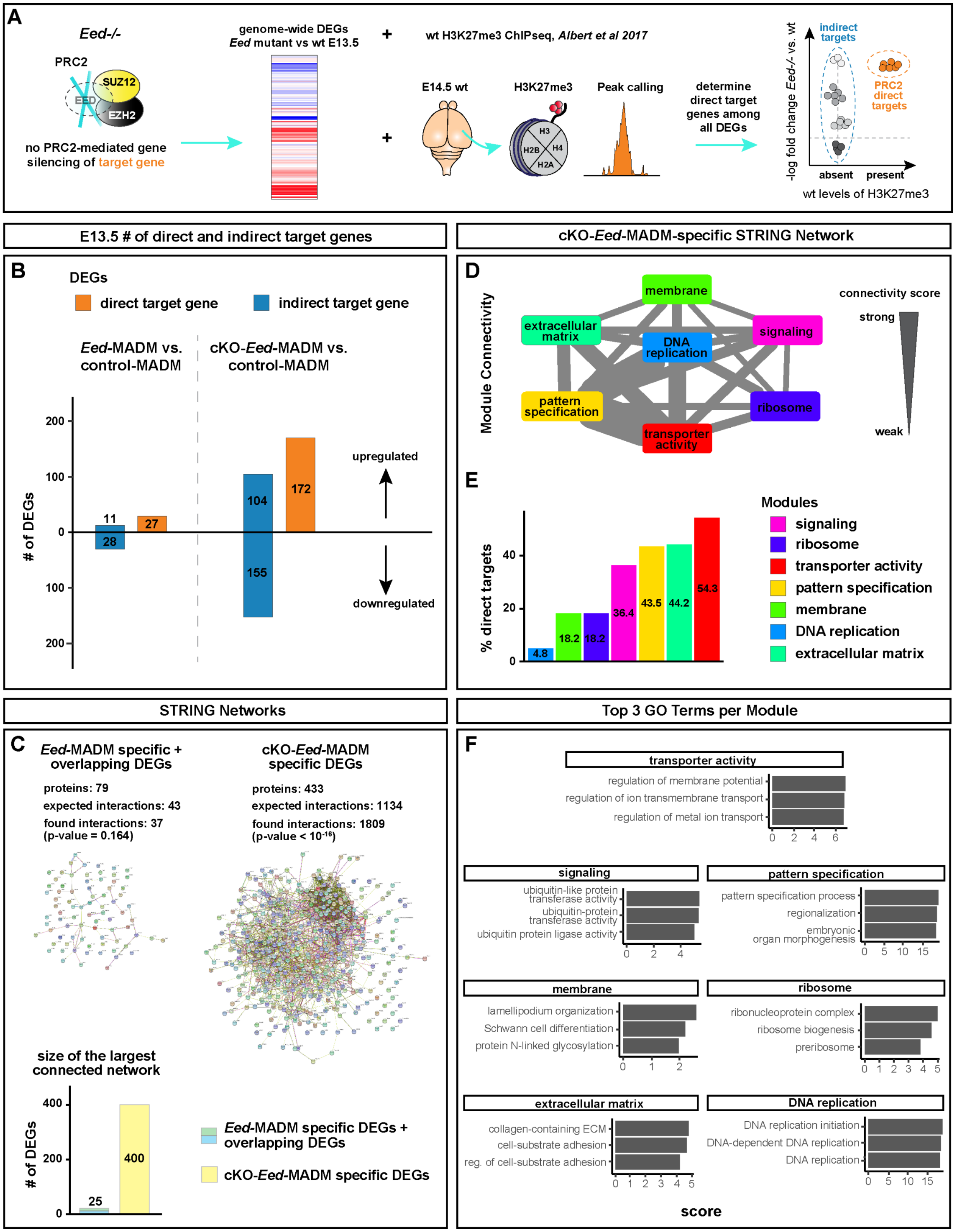
A
Eed-/-
PRC2
SUZ12
EED
EZH2
no PRC2-mediated gene silencing of target gene
genome-wide DEGs
Eed mutant vs wt E13.5
+
wt H3K27me3 ChIPseq, Albert et al 2017
E14.5 wt
H3K27me3
Peak calling
H3
H4
H2B
H2A
determine direct target genes among all DEGs
-log fold change Eed-/- vs. wt
indirect targets
PRC2 direct targets
absent
present
wt levels of H3K27me3
E13.5 # of direct and indirect target genes
B
DEGs
direct target gene
indirect target gene
Eed-MADM vs. control-MADM
cKO-Eed-MADM vs. control-MADM
# of DEGs
200
100
0
100
200
11
28
27
104
155
172
upregulated
downregulated
cKO-Eed-MADM-specific STRING Network
D
Module Connectivity
membrane
extracellular matrix
DNA replication
signaling
pattern specification
ribosome
transporter activity
connectivity score
strong
weak
E
% direct targets
40
20
0
4.8
18.2
18.2
36.4
43.5
44.2
54.3
Modules
signaling
ribosome
transporter activity
pattern specification
membrane
DNA replication
extracellular matrix
STRING Networks
C
Eed-MADM specific + overlapping DEGs
proteins: 79
expected interactions: 43
found interactions: 37
(p-value = 0.164)
cKO-Eed-MADM specific DEGs
proteins: 433
expected interactions: 1134
found interactions: 1809
(p-value < 10-16)
size of the largest connected network
# of DEGs
400
200
0
25
400
Eed-MADM specific DEGs + overlapping DEGs
cKO-Eed-MADM specific DEGs
Top 3 GO Terms per Module
F
transporter activity
regulation of membrane potential
regulation of ion transmembrane transport
regulation of metal ion transport
0 2 4 6
signaling
ubiquitin-like protein transferase activity
ubiquitin-protein transferase activity
ubiquitin protein ligase activity
0 2 4
pattern specification
pattern specification process
regionalization
embryonic organ morphogenesis
0 5 10 15
membrane
lamellipodium organization
Schwann cell differentiation
protein N-linked glycosylation
0 1 2
ribosome
ribonucleoprotein complex
ribosome biogenesis
preribosome
0 1 2 3 4 5
extracellular matrix
collagen-containing ECM
cell-substrate adhesion
reg. of cell-substrate adhesion
0 1 2 3 4 5
DNA replication
DNA replication initiation
DNA-dependent DNA replication
DNA replication
0 5 10 15
score

Figure S5. Related to Figure 3. Identification and STRING network analysis of direct and indirect deregulated PRC2 target genes in *Eed*$^{-/-}$ cells upon sparse and global *Eed* deletion.

(A) Strategy to determine direct PRC2 target genes using our RNAseq datasets from *Eed*$^{-/-}$ mutant cells aligned with H3K27me3 ChIPseq data from (*39*).

(B) Bar graph showing the number of differentially expressed direct and indirect PRC2 target genes in *Eed*-MADM and cKO-*Eed*-MADM. Direct targets are indicated in orange and indirect targets in blue color. DEGs are from comparison of E13.5 *Eed*$^{-/-}$ cells isolated from *Eed*-MADM versus control-MADM and *Eed*$^{-/-}$ cells isolated from cKO-*Eed*-MADM versus control-MADM.

(C) (Top) Full overview of STRING networks derived from *Eed*-MADM-specific plus overlapping DEGs and from cKO-*Eed*-MADM specific DEGs. Note that only the cKO-*Eed*-MADM specific network shows a significant enrichment of connections. (Bottom) Bar graph highlighting the number of DEGs giving rise to the largest connected network within the above STRING networks (bottom).

(D) Connectivity Score of cKO-*Eed*-MADM specific STRING network.

(E) Bar graph showing the percentage of direct target genes per individual module in the cKO-*Eed*-MADM specific STRING network.

(F) Top 3 Gene Ontology terms per STRING network module.

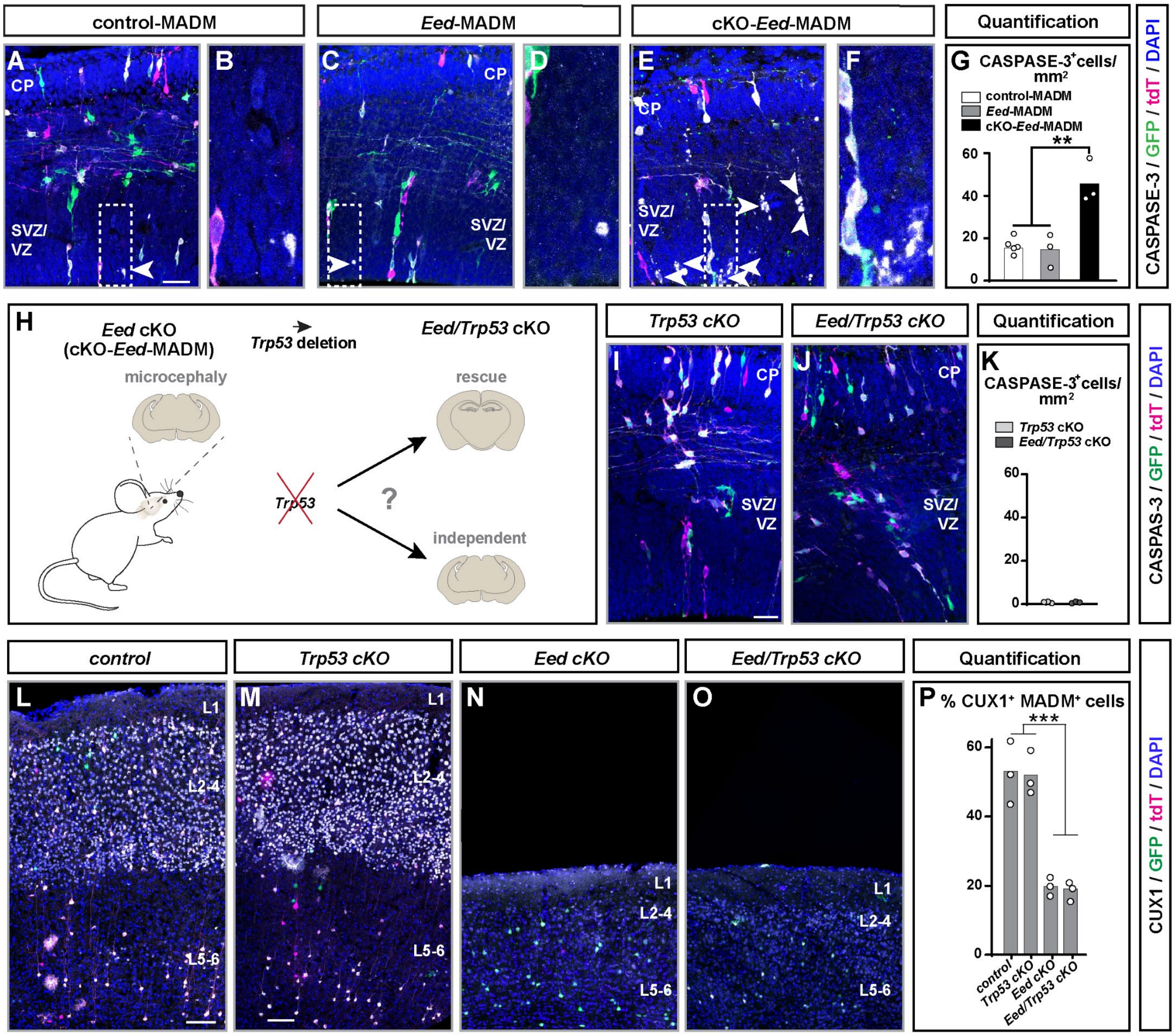

Figure S6. Related to Figure 4. Microcephaly in cKO-*Eed*-MADM develops in *Trp53*-independent manner.

(A-F) Histological stainings of CASPASE-3 in (A-B) control-MADM; (C-D) *Eed*-MADM; and (E-F) cKO-*Eed*-MADM at E14.5. (B, D, F) Higher magnification of boxed areas in (A), (C) and (E) highlighting the VZ/SVZ with apoptotic cells.

(G) Percentage of CASPASE-3^{+} cells/mm^{2} in (white) control-MADM; (grey) *Eed*-; and (black) cKO-*Eed*-MADM.

(H) Experimental paradigm to test the possible role of *Trp53* in the emergence of microcephaly phenotype upon global KO of *Eed* in cKO-*Eed*-MADM.

(I-J) Histological stainings of CASPASE-3 in (I) *Trp53* cKO and (J) *Eed*/*Trp53* double cKO at E14.5.
(K) Number of CASPASE-3^{+} cells/mm^{2} in *Trp53* cKO (light grey) and *Eed*/*Trp53* double cKO (dark grey) at E14.5.
(L-O) Confocal images with layer indications depicting immunofluorescence stainings for upper layer marker CUX1 in brains from (L) control; (M) *Trp53* cKO; (N) *Eed* cKO; and (O) *Eed/Trp53* double cKO mice at P21.
(P) Percentage of CUX1^{+} MADM-labelled cells in brains from control, *Trp53* cKO; *Eed* cKO and *Eed/Trp53* double cKO mice at P21.
Each individual data point in (G), (K) and (P) represents one experimental animal. Data indicate mean ± SEM. Statistics: (G, P) one-way ANOVA with Turkey's multiple comparisons; * $p<0.05$; ** $p<0.01$; *** $p<0.001$. (K) unpaired t-test; * $p<0.05$; ** $p<0.01$; *** $p<0.001$.
Scale bars: 25μm in (A, C, E); 8μm in (B, D, F); 20μm in (I, J) and 100μm in (L-O).

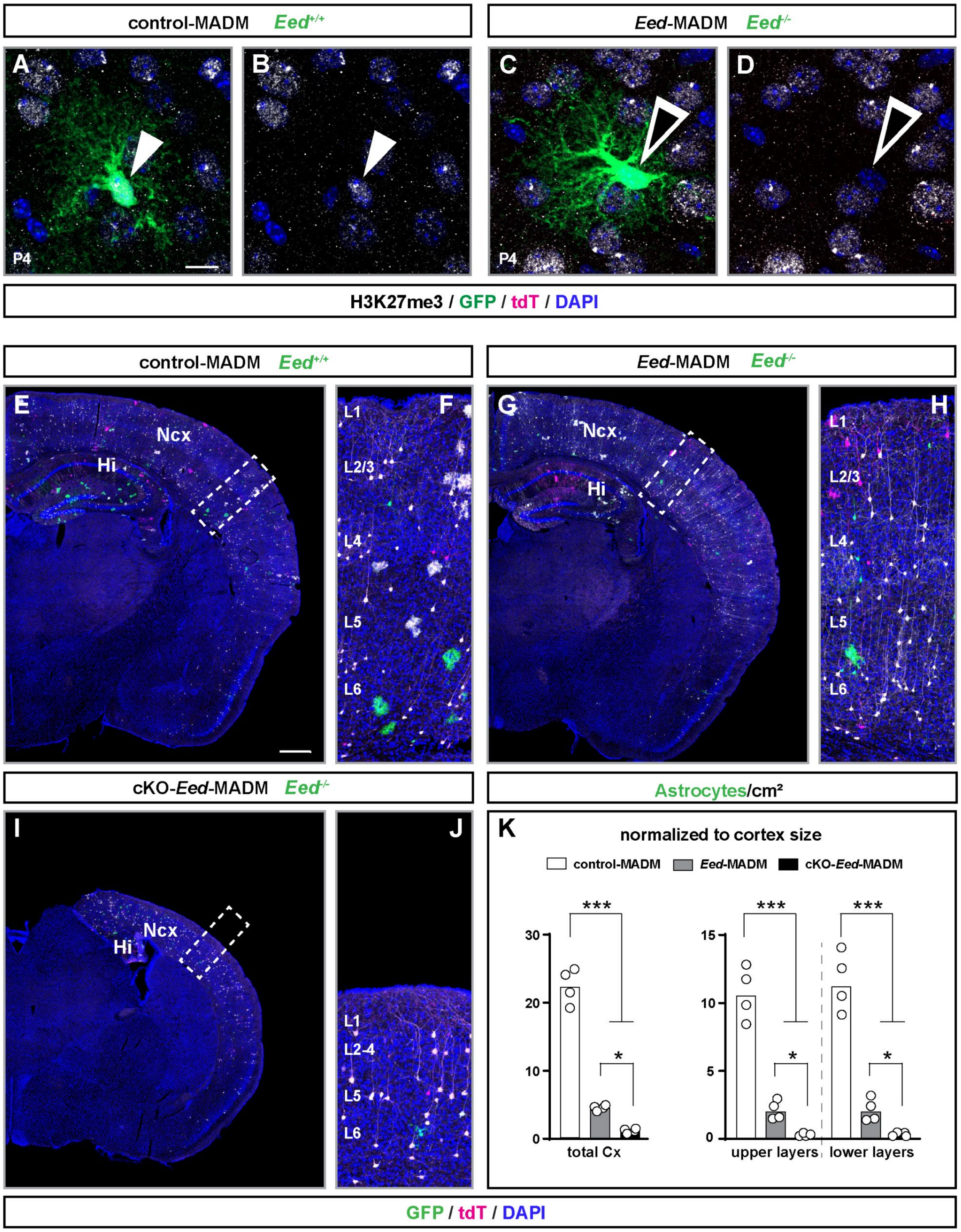

Figure S7. Related to Figure 5. Astrocyte generation upon loss of *Eed*.

(A-D) High resolution images of green GFP^{+} astrocytes in P4 (A-B) control-MADM and (C-D) *Eed*-MADM stained for H3K27me3. (B and D) Images from (A and C) showing H3K27me3 and DAPI only. Closed white arrowheads point on an immature *Eed*$^{+/+}$ astrocyte with H3K27me3

marks. Black arrowheads with white lining point on an immature *Eed*$^{-/-}$ astrocyte devoid of the H3K27me3 marks.

(E-J) Overview of MADM-labeling pattern in somatosensory cortex in (E) control-MADM; (G) *Eed*-MADM; and (I) cKO-*Eed*-MADM mice at P21. (F, H, J) depict higher resolution images of boxed areas in (E), (G) and (I) with indication of cortical layers.

(K) Quantification of number of GFP^{+} astrocytes normalized to cm^{2} of cortical size in control-MADM (white), *Eed*-MADM (grey) and cKO-*Eed*-MADM (black) mice. Quantification is shown as total numbers in the somatosensory cortex (left) and numbers in upper layers and lower layers (right).

Each individual data point represents one experimental animal. Data indicate mean ± SEM. Statistics: one-way ANOVA with Turkey's multiple comparisons; * $p<0.05$; ** $p<0.01$; *** $p< 0.001$.

Scale bars: 10µm in (A-D), 500µm in (E, G, I); 60µm in (F, H, J).

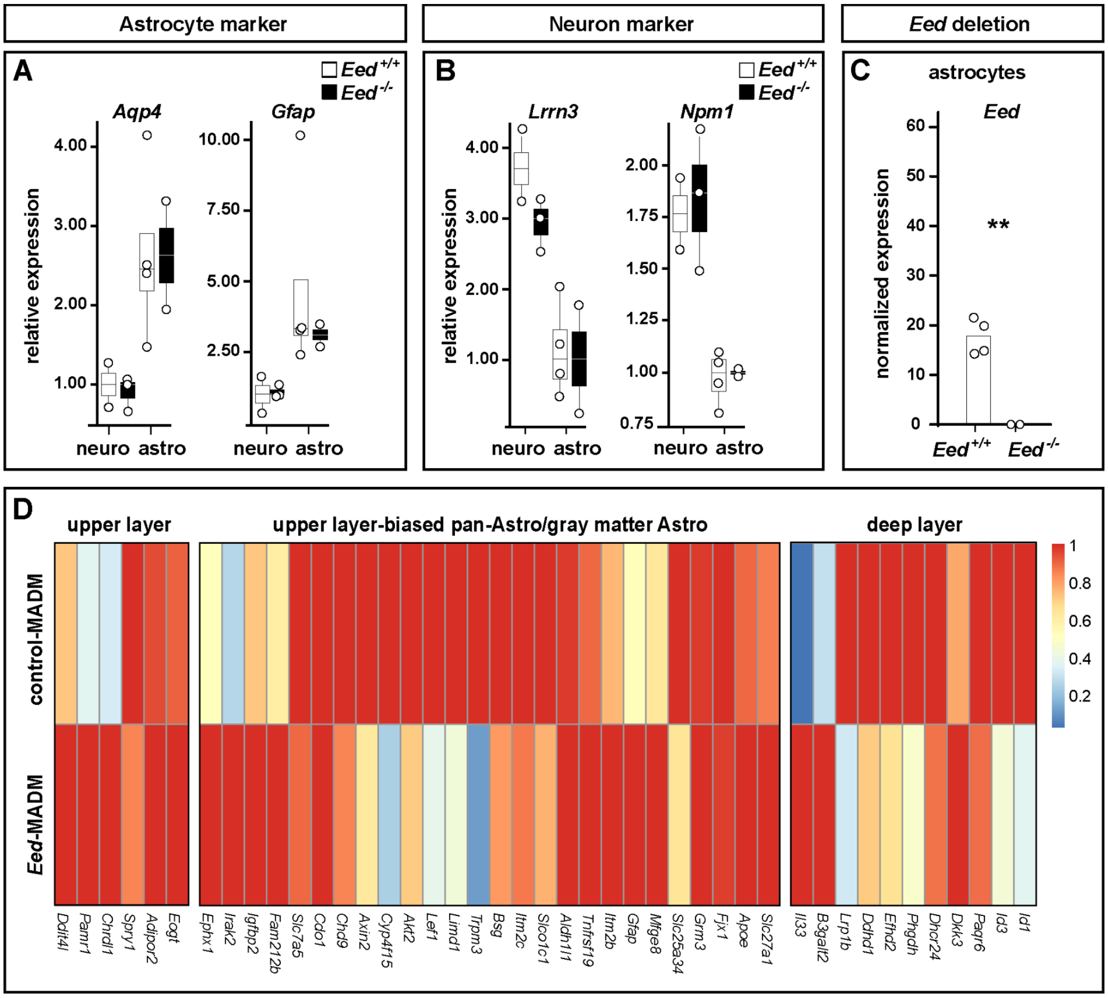

Figure S8. Related to Figure 6. PRC2 activity and gene expression upon *Eed* ablation in cortical astrocytes.

(A-B) Assessment of purity of P4 GFP$^+$/lacZ$^+$ (astrocyte) and GFP$^+$/lacZ$^-$ (neuron) cell populations by comparing the expression of (A) astrocyte marker genes *Aqp4* and *Gfap* and (B) neuron marker genes *Lrrn2* and *Npm1* in purified *Eed*$^{+/+}$ (white) and *Eed*$^{-/-}$ (black) neuron and astrocyte cell populations. Abbreviations: neuro – neurons; astro – astrocytes.

(C) Normalized *Eed* expression in purified *Eed*$^{+/+}$ (white) and *Eed*$^{-/-}$ (black) astrocytes isolated from control-MADM and *Eed*-MADM at P4.

(D) Expression of cortical astrocyte marker genes as defined in (*50*) from *Eed*$^{+/+}$ and *Eed*$^{-/-}$ astrocytes isolated at P4.

Each individual data point represents one sample derived from an individual experimental animal. Data show mean ± SEM.

Table S1. Key resources table.

Table contains information about the reagents, mice, software, packages and other crucial material used in the presented study.

REAGENT or	SOURCE	IDENTIFIER
**Chemicals, Peptides, and Recombinant Proteins**		
PFA	Sigma-Aldrich	Cat# 441244
10X PBS	ThermoScientific	Cat# 70011
Triton X-100 for Histology	Sigma-Aldrich	Cat# T878
Tween 20	Sigma-Aldrich	Cat# P9416
0.2M Tris HCL pH 8.5	Roth	Cat# 9090
$NaH_2PO_4 \cdot 2H_2O$	Sigma-Aldrich	Cat# 567549
Na_2HPO_4	Sigma-Aldrich	Cat# S3264
Glycerol	Sigma-Aldrich	Cat# G5516
Citric Acid	Roth	Cat# X863.1
NaOH	Roth	Cat# T198.1
Heat-inactivated Horse Serum (HS)	Thermo Fisher Scientific	Cat#26050088
Heat-inactivated Fetal Bovine Serium (FBS)	Thermo Fisher Scientific	Cat#10082147
Avertin (2,2,2 triboromoethanol)	Sigma-Aldrich	Cat# T48402
t-amylalcohol (2-methyl-2-butanol)	Sigma-Aldrich	Cat# 240486
Ethanol	Honeywell	Cat# 24194
Sucrose	Sigma-Aldrich	Cat# S8501
O.C.T. Tissue Tek	Sakura	Cat# 4583
Mowiol 4-88	Roth	Cat# 0713
DABCO	Roth	Cat# 0718
Papain Vial Source	Worthington	Cat#PAP2
DNase Vial Source	Worthington	Cat#D2
Inhibitor Vial Source	Worthington	Cat#OI-BSA
EBSS	Thermo Fisher Scientific	Cat#24010043
DMEM/F12	Thermo Fisher Scientific	Cat#21041025
DAPI	Molecular Probes	Cat# D1306
Triton X-100 for RNAseq	Sigma-Aldrich	Cat#T878

RNase Inhibitor	Takara	Cat#2313A
RNase Away	Thermo Fisher Scientific	Cat#10328011
RNase free water	Thermo Fisher Scientific	Cat#10977035
1M Tris pH 8.0	Thermo Fisher Scientific	Cat# AM9855G
0.5M EDTA pH 8.0	Thermo Fisher Scientific	Cat# AM9260G
10% SDS	Thermo Fisher Scientific	Cat# AM9822
Trizol	ambion	Cat# 15596018
Chloroform	Thermo Fisher Scientific	Cat# C2432-4x25ML
Isopropanol	Thermo Fisher Scientific	Cat# I9516-4x25ML
Ethanol for RNA purification	Fisher BioReagents	Cat# BP2818-100
GlycoBlue	Thermo Fisher Scientific	Cat# AM9515
**Antibodies**		
Chicken anti-GFP antibody	Aves, dilution 1:400	RRID:AB_10000240
Goat anti-mCherry antibody	SICgen, dilution 1:400	RRID:AB_2333092
Rabbit anti-CTIP2	Abcam, dilution 1:400	RRID:AB_2064130
Goat anti-CUX1	Santa Cruz, dilution 1:100	RRID:AB_2087003
Rabbit anti-caspase 3	Cell Signaling, Dilution 1:500	RRID:AB_2341188
Rabbit anti-H3K27me3	Diagenode, Dilution 1:1,00	RRID:AB_2753161
Goat anti-SOX2	Santa Cruz, Dilution 1:500	RRID:AB_2286684
Rat anti-TBR2	ThermoScientific, Dilution 1:500	RRID:AB_11042577
Rabbit anti-NEUROD2	Abcam, Dilution 1:200	RRID:AB_10866309
Donkey anti-chicken-FITC secondary antibody	Invitrogen, dilution 1:500	RRID:AB_923386

Donkey anti-goat Alexa568 secondary antibody	Molecular Probes, dilution 1:1,000	RRID:AB_2534104
Donkey anti-goat Alexa647 secondary antibody	Molecular Probes, dilution 1:1,000	RRID:AB_141844
Donkey anti-rabbit Alexa647 secondary antibody	Molecular Probes, dilution 1:1,000	RRID:AB_2762835
Donkey anti-rat Alexa568 secondary antibody	Molecular Probes, dilution 1:1,000	RRID: AB_2910653
Donkey anti-goat Alexa488 secondary antibody	Molecular Probes, dilution 1:1,000	RRID: AB_2534102
**Critical Commercial Assays**		
FACS Blue LacZ beta Galactosidase detection kit	Abcam	Cat#ab189815
Click-iT Alexa Fluor 647 imaging kit	Thermo Fisher Scientific	Cat#C10340
Bioanalyzer Pico RNA Kit	Agilent	Cat# 5067-1513
**Experimental Models: Organisms/Strains**		
Mouse: *MADM-7-GT*	The Jackson Laboratory	RRID:IMSR_JAX:021457
Mouse: *MADM-7-TG*	The Jackson Laboratory	RRID:IMSR_JAX:021458
Mouse: *Emx1-Cre*	The Jackson Laboratory	RRID:IMSR_JAX:005628
Mouse: *Emx1-Cre*^{ER}	The Jackson Laboratory	RRID:IMSR_JAX:027784
Mouse: *Eed*-flox	The Jackson Laboratory	RRID:IMSR_JAX:022727
Mouse: *Trp53*-flox	The Jackson Laboratory	RRID:IMSR_JAX:008462
Mouse: *Trp53*-null	The Jackson Laboratory	RRID:IMSR_JAX:002101
Mouse: *hGFAP-lacZ*	The Jackson Laboratory	RRID: IMSR JAX: 003487
**Software and Algorithms**		
ZEN blue	Zeiss	http://www.zeiss.com/microscopy/en_us/products/microscope-software/zen.html#introduction

Photoshop	Adobe	adobe.com/products/photoshop
GraphPad Prism	GraphPad	https://www.graphpad.com/scientific-software/prism/
IMARIS 9.2.4	Bitplane	https://imaris.oxinst.com/products/imaris-for-neuroscientists
ImageJ	NIH	https://imagej.nih.gov/ij/index.html
STAR v2.5.0c	(*66*)	https://github.com/alexdobin/STAR
Bedtools v2.26.0	(*67*)	https://github.com/arq5x/bedtools2
R v3.6.1, v4.1.2	N/A	https://www.r-project.org/
DESeq2 v1.26.0	(*68*)	http://www.bioconductor.org/
clusterProfiler v3.14.3, v4.2.2	(*71*)	http://www.bioconductor.org/
Pheatmap v1.0.12	N/A	https://CRAN.R-project.org/package=pheatmap
STRING v11	(*40*)	https://string-db.org/
Cytoscape v3.7.2, v3.8.1	(*70*)	https://cytoscape.org/
**Other**		
Embedding molds for coronal brain sections	Polysciences Inc.	Cat# 18986-1
6well plates	TPP	Cat# 92406
24well plates	TPP	Cat# 92424
Flask filters 500ml	TPP	Cat# 99505
Microfuge tubes 1.5 mL	Thermo Fisher Scientific	Cat# AM12450
50ml Centrifuge tubes	Sarstedt	Cat# 62.547.254
15ml Centrifuge tubes	Sarstedt	Cat# 62.554.502
Syringe 60ml	Kendall	Cat# 560125
Syringe 10ml Omnifix	Braun	Cat# 4617100V
Needle 20G Sterican	Braun	Cat# 4657705
0.2micron filter	Nalgene	Cat# 194-2520
Peristaltic pump	Watson Marlow	Cat# 323 S/D
Hydrophobic pen	DAKO	Cat# S2002
Petri dish	ThermoScientific	Cat# NC9565080
Slide moisture chamber black	Newcomer Supply	Cat# 68432A
Superfrost Glass Slides	ThermoScientific	Cat# J1800AMNZ
Cover slips 24x50 mm	VWR	631-0147
Dissection Tools	F.S.T.	Various forceps and scissors
Fine brush size 1	Ted Pella Inc	Cat# 11859

LSM 800 Confocal	Zeiss	N/A
Cryostat Cryostar NX70	ThermoFisher	N/A
BD Aria III	BD Biosciences	N/A
Bioanalyzer	Agilent	N/A
HiSeq2500	Illumina	N/A
AluminaSeal	Merck	Cat#Z740251-100EA
Hard-Shell PCR Plates (96-well)	Bio Rad	Cat#HSP9631
PCR tubes & caps, RNase-free, 0.2 mL (8-strip format)	Thermo Fisher Scientific	Cat#AM12230
Falcon 5 mL polystyrene round-bottom tube with 40µm cell strainer cap	Fisher Scientific	Cat#10585801
TPP TubeSpin bioreactor tubes	Merck	Cat#Z761028-180EA
Bioanalyzer Pico chips	Agilent	Cat# 5067-1513
High Density Microfuge Tubes	Qiagen	129056

Data Table S1. (separate file) Complete results of E12.5, E13.5, E16.5 and P0 DEG, E13.5 H3K27me3 and GO term enrichment analysis.

Tabs labeled with DEG contain results from DESeq2 analyses, tabs labeled with GO contain results of GO term enrichment analyses from clusterProfiler. Tab DEG.E13_cKO_wt contains additional information on STRING analysis (cluster, pred_function) and H3K27me3 analysis (column direct_H3K27me3). Column cluster indicates arbitrary numbering of clusters in the STRING network and are the same as for the labels of the GO term enrichment analyses. Abbreviations: cKO: cKO-*Eed*-MADM, het: *Eed*-MADM, wt: control-MADM

Data Table S2. (separate file) Complete results of astrocyte DEG analysis.

Table contains output from astrocyte DEG analysis using DESeq2.

Data Table S3. (separate file) Statistics table.

Table contains the number of mice, sections, quantified cells and parameters for all figures.

Data Table S4. (separate file) Source data table.

Table contains the raw data for all figures.
